# Supplementary figures and images for: Outcomes of a 12-week ecologically valid observational study of first treatment with methylphenidate in a representative clinical sample of drug naïve children with ADHD
Source: PLoS One. 2021 Oct 21;16(10):e0253727. doi: 10.1371/journal.pone.0253727 (PMC8530346; doi:10.1371/journal.pone.0253727)

**S1 Fig.**

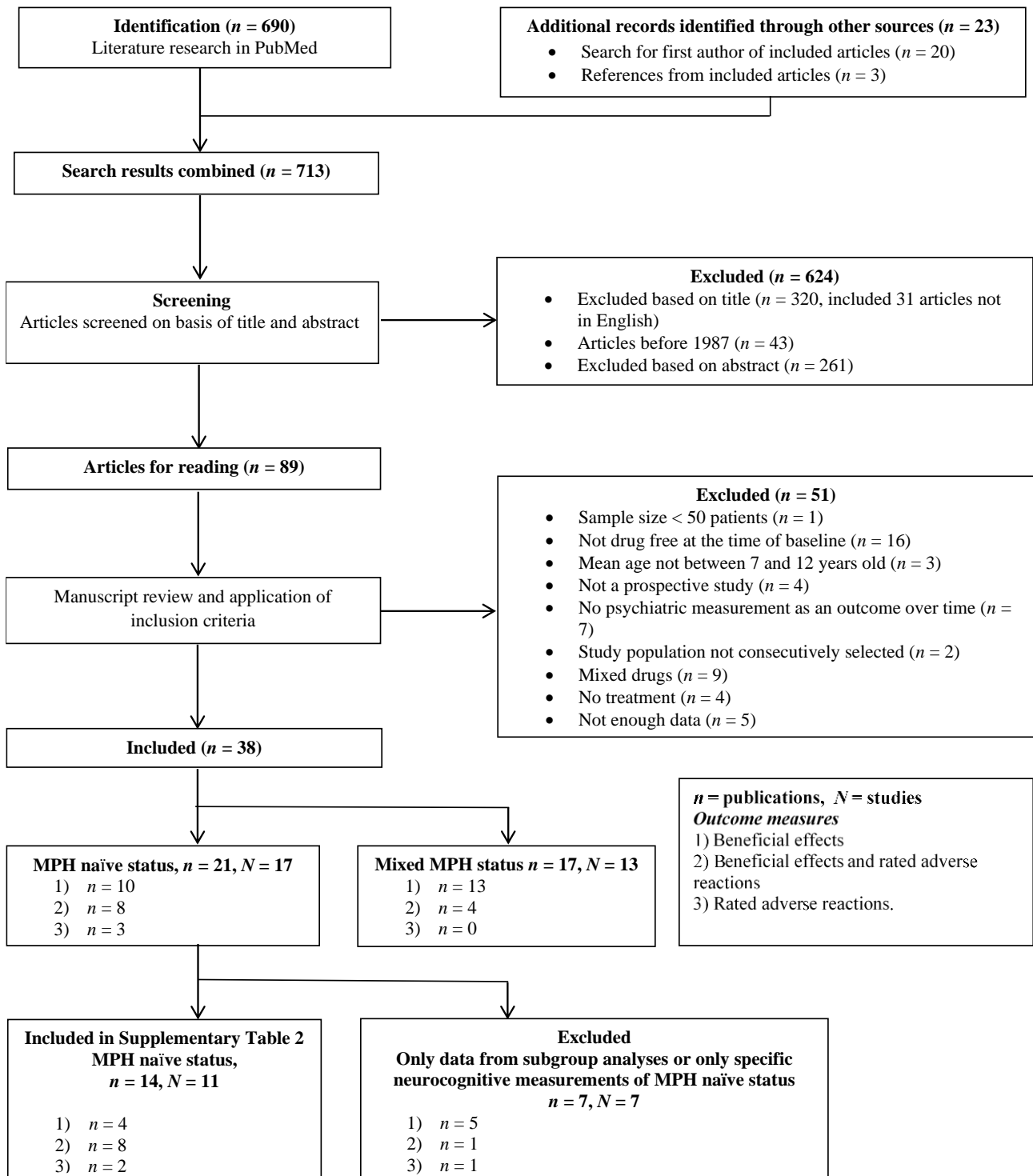

Supplement: S1 Fig — (PDF) [file pone.0253727.s001.pdf]
