# Supplementary material for: Outcomes of a 12-week ecologically valid observational study of first treatment with methylphenidate in a representative clinical sample of drug naïve children with ADHD
Source: PLoS One. 2021 Oct 21;16(10):e0253727. doi: 10.1371/journal.pone.0253727 (PMC8530346; doi:10.1371/journal.pone.0253727)
Supplement: S1 Table — (PDF) [file pone.0253727.s002.pdf]

S1 Table. Criteria for literature search

Inclusion criteria for articles:

1) Sample size above 50 patients. No upper limit.
2) Mean or median age between 7 and 12 years old.
3) ADHD/ADD diagnosed as *DSM*-IIIR, *DSM*-IV, *DSM*-5 or ICD-10
4) Prospective longitudinal cohort studies with a psychiatric measurement as an outcome over time.
5) Treatment with methylphenidate as tablets. A group of patients should be treated only with MPH and the MPH should reported separately.
6) Consecutively selected patients from child and adolescent, pediatric departments or from a specific geographic area.
7) A group of patients should be methylphenidate-free[1] at baseline.

Exclusion criteria for articles:

1) Epidemiological studies using register-based data for prescriptions and/or diagnosis.
2) Only subgroups of patients with ADHD and a specific comorbidity.
3) Studies which only measure treatment outcomes after switching of medication.
4) Reviews, case reports and randomized controlled trials.
5) Articles before 1987.

DSM = Diagnostic and Statistical Manual of Mental Disorders, ICD-10 = International Classification of Diseases and Related Health Problems
[1] = No methylphenidate treatment at the time of inclusion
