# Supplementary material for: Outcomes of a 12-week ecologically valid observational study of first treatment with methylphenidate in a representative clinical sample of drug naïve children with ADHD
Source: PLoS One. 2021 Oct 21;16(10):e0253727. doi: 10.1371/journal.pone.0253727 (PMC8530346; doi:10.1371/journal.pone.0253727)
Supplement: S2 Table — (PDF) [file pone.0253727.s003.pdf]

S2 Table. Included studies in the review of naturalistic observational clinical prospective studies of MPH treatment of drug naïve children with ADHD

**Studies**<br><br>**Study number (References)**	**Total included *N* Boys, *n* (%)**	**Mean age (SD)**	**ADHD subtype *DSM*-IV**<br><br>***N* (%)**	**FU after initiation of MPH, weeks**	**End-dose of MPH mg/kg/day (SD)**	**Response definitions**	**Response rates**	**Calculated absolute mean reduction score of ADHD core symptoms in %**
**1:** *Kim B-N et al.* 2011(1) *	*N* = 102<br>*n* = 82 (80.4)	8.7 (2.1)	27 ADHD-I (26.4%)<br>6 ADHD-HI (5.8%)<br>69 ADHD-C (67.6%)	2, 4 8, 12	0.98 (0.50)	1: Sum score ≤ 18 and single item score ≤ 1 on ADHD-RS-C (1-18), and ≤ 2 on CGI-I<br>2: ≤ 2 on CGI-S<br>3: ≥ 50% decrease on ADHD-RS-C (1-18)	1: (*n* = 80) 78.4%<br>2: (*n* = 47) 46.1%<br>3: (*n* = 22) 21.6%	ADHD-RS-C (1-18); 62.7%
**2:** *Lee et al.* 2011(2) *	*N* = 137<br>Completed, *N* = 112<br>*n* = 93 (83.0)	*N* = 112<br>10.2 (2.9)	21 ADHD-I (18.8%)<br>1 ADHD-HI (0.9%)<br>90 ADHD-C (80.4%)	1, 2, 4, 8	Responder<br>*n* = 76: 0.85 (0.27) (67.9%)<br>Non-responder<br>*n* = 36: 0.86 (0.30) (32.1%),<br>*n* = 112: 0.85	≥ 50% decrease on ADHD-RS-P (1-18) and ≤ 2 on CGI-I	(*n* = 76) 67.9%	ADHD-RS-P (1-18); 38.5%
**3:** *Johnson et al.* 2013(3) *	*N* = 77<br>*n* = 66 (85.7)	8.0 (2.6)	7 ADHD-I (9%),<br>7 ADHD-HI (9%),<br>63 ADHD-C (82%).	6	0.57 (0.19)<br>0.54 (0.20) (IR-MPH)	No definition of response to MPH (CPRS-R:S ADHD index T-score)		CPRS-R:S; 23.8%
**4:** *Garcia et al.* 2009(4)	*N* = 280<br>*n* = 211 (75.4)	ADHD+ANX: *N* = 76: 9.9 (3.0)<br>ADHD-ANX: *N* = 204: 10.2 (3.0)<br>ANX=Anxiety<br>*n* = 280: 10.0	No information about ADHD sub-types.	4	ADHD+ANX<br>*n* = 76 (27.1%): 0.45 (0.14).<br>ADHD-ANX,<br>*n* = 204 (72.9%): 0.52 (0.16),<br>*n* = 280: 0.50	≥ 50% decrease on SNAP-IV	(*n* = 88) 31.4%	Not possible
**5:** *Paton et al.* 2014(5)	*N* = 51<br>*n* = 43 (84.3)	8.4 (2.4)	4 ADHD-I (8%)<br>3 ADHD-HI (6%)<br>44 ADHD-C (86%)	6	0.58 (0.18)	No definition of response to MPH		CPRS-R:S; 24.4%
**6:** *Song et al.* 2014(6) *	*N* = 139<br>*n* = 120 (86.3)	10.0 (2.8)	28 ADHD-I (20.1%)<br>3 ADHD-HI (2.2%)<br>102 ADHD-C (73.9%)<br>6 ADHD-NOS (4.3%)	8	31.0 (9.0) mg/day<br><br>No weight available.	1: ≥ 50% decrease on ADHD-RS-P (1-18)<br>2: ≤ 2 on CGI-I<br>3: ≥ 50% decrease on ADHD-RS-P (1-18) and ≤ 2 on CGI-I	1: (*n* = 99) 71.2%<br>2: (*n* = 110) 79.1%<br>3: (*n* = 90) 64.7%	Not possible
**7:** *Kim JI et al.* 2016(7) *	*N* = 75<br>*n* = 64 (85.3)	8.8 (2.2)	24 ADHD-I (32%)<br>3 ADHD-HI (4%)<br>39 ADHD-C (52%)<br>9 ADHD-NOS (12%)	(2), (4), (6), 8, 16, 24. (limited data of these weeks)	28.1 (12.3) mg/day<br><br>No weight available	1: ≥ 40% decrease on ADHD-RS-P (1-18)<br>2: ≤ 2 on CGI-I<br>3: ≥ 40% decrease on ADHD-RS-P (1-18) and ≤ 2 on CGI-I.	1: (*n* = 38) 50.7%<br>2: (*n* = 37) 49.3%<br>3: (*n* = 19) 25.3%	ADHD-RS-P (1-18); 41.8%
**8:** *Park et al.* 2013(8)<br>*Kim J-W et al* 2013(9)	*N* = 132<br>*n* = 108 (81.8)	8.8 (1.5)	N = 131 (99.2%)<br>39 ADHD-I (29.5%)<br>6 ADHD-HI (4.5%)<br>78 ADHD-C (59.1%)<br>8 ADHD-NOS (6.1%)	2, 4, 8, 12	1.00 (0.28)	1: ≥ 50% decrease on ADHD-RS-P (1-18) and no response on TOVA<br>2: ≤ 15% decrease on mean composite score of TOVA and in 1<br>3: No response in 1 and 2<br>4: Response in 1 and 2.<br>1: ≤ 18 ADHD-RS-C (1-18) and ≤ 2 CGI-I	1: (*n* = 29) 24.4%<br>2: (*n* = 18) 15.1%<br>3: (*n* = 43) 36.1%<br>4: (*n* = 29) 24.4%<br>1: (*n* = 101) 76.5%	Not possible<br>ADHD-RS-C (1-18): 62.2%<br>ADHD-RS-C (1-9): 59.3%<br>ADHD-RS-C (10-18): 66.0%<br>ADHD-RS-P (1-18): 43.9%<br>ADHD-RS-P (1-9): 41.4%<br>ADHD-RS-P (10-18): 47.3%
**9:** *Pagerols et al.* 2018(10) *	*N* = 173<br>*n* = 147 (84.9)<br>*N* = 107	9.6 (2.9)<br>9.4 (2.8)	37 ADHD-I (21.4%)<br>5 ADHD-HI (2.9%)<br>131 ADHD-C (75.7%)	8	1.06 (0.28)<br>1.07 (0.30)	1: ≤ 2 on CGI-I<br>1: ≤ 2 on CGI-I	1: (n =141) (81.5 %)<br>1: (n = 84) (78.5 %)	No possible

Pagerols et al. 2017(11) *	*n* = 95 (88.8)		22 ADHD-I (20.6 %)<br>6 ADHD-HI (5.6 %)<br>79 ADHD-C (74.0 %)				
Study						**ARs definition**	**Adverse effect rates**
1: *Cho et al.* 2012(12)*<br>(*Kim B-N et al.* 2011(1) *)	-	-	-	-	-	No definition	No significant change in blood pressure (BP), heart rate (HR) or electrocardiography.<br>BP changed < 0.1mmHg and HR changed < 4 bpm.
2: *Lee et al.* 2011(12–15)(2) *	-	-	-	-	-	No definition	Lack of effect; 0.7% (*n* = 1)<br>Intolerable ARs; 8.0% (*n* = 11)<br>Refusal of MPH treatment; 0.7% (*n* = 1)<br>Not attending scheduled clinic visit; 8.8% (*n* = 12)
3: *Johnson et al.* 2013(3) *	-	-	-	-		BSSERS-C	Reduced appetite 66% (*n* = 50), 0.6 mg/kg/day (0.2)<br>Weight loss 34% (*n* = 26), 0.7 mg/kg/day (0.2)<br>Headache or abdominal pain; 50% (*n* = 38), irritability; 46% (*n* = 35), sadness; 38% (*n* = 29), insomnia (new onset); 33% (*n* = 25), insomnia (exacerbation); 30% (*n* = 23), tics (new onset); 3% (*n* = 2), tics (exacerbation); 7% (*n* = 5)
8: *Kim J-W et al* 2013(9)<br><br>(*Park et al.* 2013(8))	-	-	-	-	-	BSSERS-C<br><br>Not whole scale represented	Any ARs; 75.0% (n = 99)<br>Dropped out due to ARs: 4.5% (*n* = 6): insomnia (*n* = 2), reduced appetite (*n* = 1), tics (*n* = 1), rash (*n* = 1), tremor (*n* = 1).<br>Reduced appetite; 58.3% (*n* = 77), insomnia; 43.9% (*n* = 58), abdominal pain; 28.0% (*n* = 37), Anxiety; 27.3% (*n* = 36), headache; 23.5% (*n* = 31), uninterested; 22.0% (*n* = 29), nail biting; 17.4% (*n* = 23), somnolence; 15.9% (*n* = 21), incommunicative; 15.2% (*n* = 20), daydream; 14.4% (*n* = 19), nausea; 12.1% (*n* = 16), nightmare; 9.8% (*n* = 13), dizziness; 8.3% (*n* = 11)<br>Mean (SD) significant decrease: BMI Z-score of 0.3 (1.1), weight 0.9 kg (1.9)<br>Mean (SD) significant increase: pulse rate 2.8 bpm (10.8)<br>No significant difference: systolic and diastolic blood pressure.
10: *Bruxel et al.* 2013(13) *	*N* = 213<br>Completed;<br>*N* = 205<br>*n* = 151 (73.6)	*N* = 129:<br>10.4 (2.9)<br>*N* = 76:<br>10.0 (3.1)<br>*n* = 205: 10.0	N = 205<br>57 ADHD-I (27.8%)<br>14 ADHD-HI (6.8%)<br>117 ADHD-C (57.1%)<br>17 ADHD-NOS (8.3%)	4, 12		Low score, BSSERS-P = 0-5.<br>High score, BSSERS-P = 6-9.	FU appetite low score BSSERS-P; 79.7% (*n* = 94)<br>FU appetite high score BSSERS-P; 21.3% (*n* = 24)
11: *Kim et al.* 2013(14) *	*N* = 147<br>Completed;<br>*N* = 134<br>*n* = 112 (83.6)	*N* = 134<br>10.3 (2.9)	23 ADHD-I (17.2%)<br>2 ADHD-HI (1.5%)<br>102 ADHD-C (76.1%)<br>7 ADHD-NOS (5.2%)	1, 2, 4	0.86 (0.30)	BSSERS-P	1 ≥ ARs; 82.8% (*n* = 111)<br>Mean BSSERS-P; 11.3 (SD 14.1)<br>Reduced appetite; 56.7% (*n* = 76)<br>Trouble sleeping; 41.8% (*n* = 56)<br>Talking little with others; 22.4% (*n* = 30)<br>Irritability; 20.9% (*n* = 28)
9: *Pagerols et al.* 2017(11) *	-	-	-	-	-	BSSERS-C	Any ARs 65.1% (*n* = 69); insomnia 34.3% (*n* = 37), reduced appetite 25.0 % (*n* = 27).
Total<br>***Mean***<br>***Range***	**1537**<br>**139.7**<br>**(51-280)**	**102.8**<br>**9.3**<br>**(8.0-10.3)**		**104**<br>**9.5**<br>**(4-24)**	 **0.80****<br>**(0.50-1.06)**		

* = Pharmacogenetic studies. ** = Mean dose per kg per day based on 8 studies. ARs = Adverse reactions. FU = Follow up. MPH = methylphenidate, SD = standard deviation
Only data from *Pagerols et al.* 2018(10) is included in the calculation of included patients and mean of age and end-dose.
ADHD-RS (DuPaul) Single item [range 0-3]. ADHD-RS (1-9) = Inattention subscale [range 0-27], ADHD-RS (10-18) = hyperactivity-impulsivity subscale [range 0-27)], ADHD-RS (1-18) = Inattention and hyperactivity-impulsivity [range 0-54].
ADHD-RS-C = clinician rated, ADHD-RS-P = parents rated, ADHD-RS-T = teacher rated.
CGI-I: Clinical Global Impression Improvement [range 1-7], CGI-S: Clinical Global Impression Severity [range 1-7].

CPRS-R:S: Connors Parent Rating Scale Short version. 27 items, oppositional, cognitive problems/inattention hyperactivity (18 items), ADHD index score (9 items + 3 items from the 18 items) in mixed order. Single item [range 0-3]. Converted to ADHD index T-scores in week 0 and at the time of FU.
SNAP-IV: ADHD Rating scale, Swanson Nolan Pelham. 90 items. 18-20 items of ADHD. SNAP-IV 1-9 = inattention subscale, SNAP-IV 10 = summarize inattention, SNAP-IV 11-19 = hyperactivity-impulsivity subscale, SNAP-IV 20 = summarize hyperactivity-impulsivity. Single item [range 0-3].
BSSERS: Barkley's Stimulant Side Effect Rating Scale. BSSERS-P =parent rated, BSSERS-C = clinician rated.
TOVA: Test of Variables of Attention, Composite score = Mean score of the four domains: Response time, variability of response time, omission errors, and commission errors.
ADHD *DSM-IV*:

- 314.01 Combined subtype = ADHD-comb subtype = ADHD-C subtype
- 314.01 Predominantly hyperactive-impulsive subtype = ADHD-HI subtype
- 314.00 Predominantly inattentive subtype = ADHD-I subtype
- 314.9 Attention-Deficit Hyperactivity Disorder Not Otherwise Specified = ADHD-NOS

Calculated absolute mean reduction score = (sum score of ADHD core symptoms in week 0 - sum score of ADHD core symptoms at time of FU) / sum score of ADHD core symptoms in week 0 in %.

References

1. Kim B-N, Cummins TDR, Kim J-W, Bellgrove MA, Hong S-B, Song S-H, et al. Val/Val genotype of brain-derived neurotrophic factor (BDNF) Val^{66}Met polymorphism is associated with a better response to OROS-MPH in Korean ADHD children. Int J Neuropsychopharmacol. 2011 Nov;14(10):1399–410.
2. Lee SH, Kim SW, Lee MG, Yook K-H, Greenhill LL, Fradin KN, et al. Lack of association between response of OROS-methylphenidate and norepinephrine transporter (SLC6A2) polymorphism in Korean ADHD. Psychiatry Res. 2011 Apr 30;186(2–3):338–44.
3. Johnson KA, Barry E, Lambert D, Fitzgerald M, McNicholas F, Kirley A, et al. Methylphenidate side effect profile is influenced by genetic variation in the attention-deficit/hyperactivity disorder-associated CES1 gene. J Child Adolesc Psychopharmacol. 2013 Dec;23(10):655–64.
4. Garcia SP, Guimarães J, Zampieri JF, Martinez AL, Polanczyk G, Rohde LA. Response to methylphenidate in children and adolescents with ADHD: does comorbid anxiety disorders matters? J Neural Transm Vienna Austria 1996. 2009 May;116(5):631–6.
5. Paton K, Hammond P, Barry E, Fitzgerald M, McNicholas F, Kirley A, et al. Methylphenidate improves some but not all measures of attention, as measured by the TEA-Ch in medication-naïve children with ADHD. Child Neuropsychol J Norm Abnorm Dev Child Adolesc. 2014;20(3):303–18.
6. Song J, Kim SW, Hong HJ, Lee MG, Lee BW, Choi TK, et al. Association of SNAP-25, SLC6A2, and LPHN3 with OROS methylphenidate treatment response in attention-deficit/hyperactivity disorder. Clin Neuropharmacol. 2014 Oct;37(5):136–41.
7. Kim JI, Kim J-W, Park J-E, Park S, Hong S-B, Han DH, et al. Association of the GRIN2B rs2284411 polymorphism with methylphenidate response in attention-deficit/hyperactivity disorder. J Psychopharmacol Oxf Engl. 2016 Sep 13;
8. Park S, Kim B-N, Cho S-C, Kim J-W, Shin M-S, Yoo H-J, et al. Baseline severity of parent-perceived inattentiveness is predictive of the difference between subjective and objective methylphenidate responses in children with attention-deficit/hyperactivity disorder. J Child Adolesc Psychopharmacol. 2013 Aug;23(6):410–4.
9. Kim J-W, Park S, Kim B-N, Shin M-S, Cho S-C, Kim J-H, et al. Parental perceived benefits of OROS-methylphenidate treatment for the child with attention-deficit/hyperactivity disorder and for parents themselves. Pharmacopsychiatry. 2013 Jun;46(4):137–46.
10. Pagerols M, Richarte V, Sánchez-Mora C, Rovira P, Soler Artigas M, Garcia-Martínez I, et al. Integrative genomic analysis of methylphenidate response in attention-deficit/hyperactivity disorder. Sci Rep. 2018 30;8(1):1881.
11. Pagerols M, Richarte V, Sánchez-Mora C, Garcia-Martínez I, Corrales M, Corominas M, et al. Pharmacogenetics of methylphenidate response and tolerability in attention-deficit/hyperactivity disorder. Pharmacogenomics J. 2017;17(1):98–104.

12. Cho S-C, Kim B-N, Cummins TDR, Kim J-W, Bellgrove MA. Norepinephrine transporter -3081(A/T) and alpha-2A-adrenergic receptor MspI polymorphisms are associated with cardiovascular side effects of OROS-methylphenidate treatment. J Psychopharmacol Oxf Engl. 2012 Mar;26(3):380–9.
13. Bruxel EM, Salatino-Oliveira A, Genro JP, Zeni CP, Polanczyk GV, Chazan R, et al. Association of a carboxylesterase 1 polymorphism with appetite reduction in children and adolescents with attention-deficit/hyperactivity disorder treated with methylphenidate. Pharmacogenomics J. 2013 Oct;13(5):476–80.
14. Kim SW, Lee JH, Lee SH, Hong HJ, Lee MG, Yook K-H. ABCB1 c.2677G>T variation is associated with adverse reactions of OROS-methylphenidate in children and adolescents with ADHD. J Clin Psychopharmacol. 2013 Aug;33(4):491–8.
