# Supplementary material for: Outcomes of a 12-week ecologically valid observational study of first treatment with methylphenidate in a representative clinical sample of drug naïve children with ADHD
Source: PLoS One. 2021 Oct 21;16(10):e0253727. doi: 10.1371/journal.pone.0253727 (PMC8530346; doi:10.1371/journal.pone.0253727)
Supplement: S4 Table — (PDF) [file pone.0253727.s005.pdf]

S4 Table. Baseline characteristics of included patients (*n* = 207)

**Parents civil status**	***n* (%)**
- Parents divorced	96 (46.4)
**Maternal education**	***n* (%)**
- Primary school (10 years)	24 (11.6)
- Short, higher education (11-14 years)	73 (35.3)
- Medium, higher education (15-16 years)	77 (37.2)
- Long, higher education (17-19 years)	26 (12.6)
- Unknown	7 (3.4)
**Paternal education**	***n* (%)**
- Primary school (10 years)	35 (16.9)
- Short, higher education (11-14 years)	47 (22.7)
- Medium, higher education (15-16 years)	65 (31.4)
- Long, higher education (17-19 years)	38 (18.4)
- Unknown	22 (10.6)
**Ethnicity of parents**	***n* (%)**
- Both parents of Nordic origin	190 (91.8)
- Both parents of non-Nordic origin	3 (1.4)
- One parent of Nordic origin, one parent of non-Nordic origin	14 (6.8)
**Adopted patients**	***n* (%)**
- Adopted	5 (2.4)
**Child Behaviour Check List. Parent rated.**	**M (SD)**
- CBCL externalizing score	17.9[1] (10.7)
- CBCL internalizing score	11.4[1] (7.6)
- **CBCL Total problem score**	**56.0[1] (26.2)**
- **CBCL Total T-score**	**79.6[1] (19.1)**
**Teacher Report Form. Teacher rated.**	**M (SD)**
- TRF externalizing score	23.8[2] (13.9)
- TRF internalizing score	9.6[2] (6.8)
- **TRF Total problem score**	**68.7[2] (29.0)**
- **TRF Total T-score**	**80.6[2] (18.1)**
**WFIRS-P. Parent rated.**	**M (SD)**
- WFIRS-P, Family	1.0[3] (0.7)
- WFIRS-P, School	1.0[3] (0.5)
- WFIRS-P, Daily life	0.8[4] (0.5)
- WFIRS-P, Self-perception	1.0[4] (0.8)
- WFIRS-P, Social life	1.0[4] (0.7)
- WFIRS-P, Risk behaviour	0.4[4] (0.3)
- **WFIRS-P, Total**	**0.8[4] (0.4)**

M = mean, SD = Standard deviation, *n* = number.
Number of participants with observed outcome data: [1] *n* = 147, [2] *n* = 143, [3] *n* = 195, [4] *n* = 193.
Child Behaviour Check List (CBCL). Parent rated. Externalizing score: 35 items [range 0-70]. Internalizing score: 32 items [range 0-64]. Total problem score: 118 [range 0-236]. Teacher Report Form (TRF). Teacher rated. Externalizing score, 34 items [range 0-68]. Internalizing score: 34 items [range 0-68]. Total problem score: 118 [range 0-236].
Weis Functional Impairment Rating Scale, parent version (W-FIRS-P). Family, school daily life, and risk behaviour: 10 items [range 0-30]. Self-perfection: 3 items [range 0-9]. Social life: 7 items [range 0-21]. Each subscale of WFIRS-P is divided by the number of items.
