# Supplementary material for: Outcomes of a 12-week ecologically valid observational study of first treatment with methylphenidate in a representative clinical sample of drug naïve children with ADHD
Source: PLoS One. 2021 Oct 21;16(10):e0253727. doi: 10.1371/journal.pone.0253727 (PMC8530346; doi:10.1371/journal.pone.0253727)
Supplement: S5 Table — (PDF) [file pone.0253727.s006.pdf]

S5 Table. Distribution of number of patients with normalisation and borderline normalisation of clinician rated ADHD core symptoms score in week 0 and 12 (*n* = 187)

**Inattention subscale**				
	**Week 0**<br><br>*n* (%)	**Week 12 Normalisation**<br><br>*n* (%)	**Week 12 Borderline normalisation**<br>*n* (%)	**Week 12 No normalisation or borderline normalisation**<br>*n* (%)
**Normalisation** *n* (%)	**1** (0.5)	**1**	**0**	**0**
**Borderline normalisation** *n* (%)	**3** (1.6)	**2**	**1**	**0**
**No normalisation or borderline normalisation** *n* (%)	**183** (97.9)	**67**	**66**	**50**
**Total week 12** *n* (%)	**187** (100)	**70** (37.4)	**67** (35.8)	**50** (26.7)
**IR-MPH, mg/kg/day, week 12 M** (SD)		**1.0** (0.3)	**1.0** [1] (0.3)	**1.1**[2] (0.3)
**Hyperactivity-Impulsivity subscale**				
	**Week 0**<br><br>*n* (%)	**Week 12 Normalisation**<br><br>*n* (%)	**Week 12 Borderline normalisation**<br>*n* (%)	**Week 12 No normalisation or borderline normalisation**<br>*n* (%)
**Normalisation** *n* (%)	**10** (5.3)	**10**	**0**	**0**
**Borderline normalisation** *n* (%)	**17** (9.1)	**17**	**0**	**0**
**No normalisation or borderline normalisation** *n* (%)	**160** (85.6)	**68**	**62**	**30**
**Total week 12** ***n*** (%)	**187** (100)	**95** (50.8)	**62** (33.2)	**30** (16.0)
**IR-MPH, mg/kg/day week 12** M (SD)		**1.0** (0.3)	**1.0**[3] (0.3)	**1.1** (0.3)

n = number, M = mean. SD = Standard deviation. Number of participants with observed outcome data: [1] *n* = 66, [2] *n* = 48, [3] *n* = 59.
ADHD-Rating Scale, clinician rated (ADHD-RS-C, DuPaul). Inattention subscale: 9 items [range 0-27]. Hyperactivity-impulsivity subscale: 9 items [range 0-27]. Normalisation (*t-score* ≤ 60), borderline normalisation (*t-score* ≤ 70), no normalisation or borderline normalisation of ADHD cores symptoms (ADHD-RS) due to Danish norms of sex and age.
In the article normalisation (Nor) and borderline normalisation (Bnor) on ADHD-RS-C are multiplied to Nor/Bnor = *n* (%). Number of patients who were Nor/Bnor on the Inattention subscale = 137 (73.2%) and number of patients who were Nor/Bnor on the Hyperactivity-Impulsivity subscale = 157 (84.0%).
