# Supplementary material for: Outcomes of a 12-week ecologically valid observational study of first treatment with methylphenidate in a representative clinical sample of drug naïve children with ADHD
Source: PLoS One. 2021 Oct 21;16(10):e0253727. doi: 10.1371/journal.pone.0253727 (PMC8530346; doi:10.1371/journal.pone.0253727)
Supplement: S6 Table — (PDF) [file pone.0253727.s007.pdf]

S6 Table. Absolute mean reduction score of ADHD core symptoms (*n* = 187)

Mean reduction score of ADHD core symptoms from week 0 to week 12	
**Clinician rated**	**M in % (SD)**
Inattention	51.7% (17.3)
Hyperactivity-Impulsivity	53.0% (32.0)
Inattention and Hyperactivity-Impulsivity	53.6% (15.7)
**Parent rated**	
Inattention	45.3%[1] (26.8)
Hyperactivity-Impulsivity	39.9%[2] (51.6)
Inattention and Hyperactivity-Impulsivity	45.2%[3] (24.3)
Conduct problems	43.2%[4] (52.2)
**Teacher rated**	
Inattention	31.8%[5] (28.1)
Hyperactivity-Impulsivity	[6]
Inattention and Hyperactivity-Impulsivity	[6]
Conduct problems	[6]

M = mean, SD = Standard deviation, *n* = number, *n* = number. Number of participants with observed outcome data: [1] *n* = 174, [2] *n* = 171, [3] *n* = 163, [4] *n* = 179, [5] *n* = 127, [6] = Too many single items were missing/negative sum scores to calculate.
ADHD-Rating Scale (ADHD-RS, DuPaul). Clinician rated. Inattention subscale: 9 items, range 0-27. Hyperactivity-Impulsivity subscale: 9 items [range 0-27]. Clinician rated (ADHD-RS-C), parent rated (ADHD-RS-P), and teacher rated (ADHD-RS-T).
Absolute mean reduction score = (sum score of ADHD core symptoms in week 0 - sum score of ADHD core symptoms in week 12) / sum score of ADHD core symptoms in week 0 in %.
