# Supplementary material for: Outcomes of a 12-week ecologically valid observational study of first treatment with methylphenidate in a representative clinical sample of drug naïve children with ADHD
Source: PLoS One. 2021 Oct 21;16(10):e0253727. doi: 10.1371/journal.pone.0253727 (PMC8530346; doi:10.1371/journal.pone.0253727)
Supplement: S7 Table — (PDF) [file pone.0253727.s008.pdf]

S7 Table. Further clinical follow-ups from week 0 to week 12 (*n* = 187)

	**Week 0** M (SD)	**Week 12** M (SD)	**Week 0 versus week 12**				
			**M dif. (SD)**	**95% CI**	**t (df)**	***p*-value**	**Cohen's *d***
**WFIRS-P**							
**Family**	1.0[1] (0.7)	0.7[1] (0.5)	0.3 (0.5)	(0.2, 0.3)	6.6 (167)	< 0.000	0.6
**School**	1.0[1] (0.4)	0.6[1] (0.3)	0.4 (0.4)	(0.3, 0.4)	11.5 (167)	< 0.000	1.6
**Daily life**	0.8[2] (0.5)	0.7[2] (0.4)	0.1 (0.4)	(0.0, 0.1)	2.1 (161)	0.039	0.2
**Self-perception**	1.0[2] (0.8)	0.7[2] (0.7)	0.3 (0.7)	(0.2, 0.4)	5.4 (161)	< 0.001	0.4
**Social life**	1.0[2] (0.7)	0.7[2] (0.5)	0.4 (0.5)	(0.3, 0.4)	8.9 (161)	< 0.001	0.6
**Risk behavior**	0.4[2] (0.3)	0.2[2] (0.2)	0.1 (0.2)	(0.1, 0.2)	7.4 (161)	< 0.001	0.9
**Total**	0.8[2] (0.4)	0.6[2] (0.3)	0.2 (0.3)	(0.2, 0.3)	9.5 (161)	< 0.001	0.6
**TOVA**							
**RT,** Raw score	521.3[3](113.1)	423.9[3] (89.6)	97.3 (89.8)	(80.7, 113.9)	11.6 (114)	< 0.001	1.1
**VRT,** Raw score	240.2[3] (87.4)	159.2[3] (93.0)	81.0 (76.6)	(66.9, 95.2)	11.3 (114)	< 0.001	1.1
**OE,** Raw score	47.8[3] (58.6)	12.3[3] (22.0)	35.6 (55.0)	(25.4, 45.7)	6.9 (114)	< 0.001	0.6
**CE,** Raw score	39.4[3] (26.0)	26.1[3] (20.3)	13.3 (24.1)	(8.85, 17.8)	5.9 (114)	< 0.001	0.6
**RT,** Z-score	0.0[3] (1.0)	-1.0[3] (1.0)	-1.0 (0.9)	(-1.1, -0.8)	-11.9 (114)	< 0.001	1.2
**VRT,** Z-score	0.0[3] (1.0)	-1.4[3] (1.3)	-1.4 (1.0)	(-1.5, -1.2)	-14.3 (114)	< 0.001	1.3
**OE,** Z-score	0.0[3] (1.0)	-0.9[3] (0.7)	-0.9 (0.9)	(-1.1, -0.8)	-11.6 (114)	< 0.001	2.2
**CE,** Z-score	0.0[3] (1.0)	-0.7[3] (1.03)	-0.7 (1.0)	(0.8, -0.5)	-7.1 (114)	< 0.000	0.7

Paired t-test between outcomes of week 0 and week 12. M = mean, M dif, = Mean difference, SD dif. = Standard deviation difference, *n* = number.

Number of participants with observed outcome data [1] *n* = 168, [2] *n* = 163, [3] *n* = 115.

Weiss Functional Impairment Rating Scale, parent version (W-FIRS-P). Family, school daily life, and risk behaviour: 10 items, [range 0-30]. Self-perfection: 3 items, [range 0-9]. Social life: 7 items, [range 0-21]. Each subscale of WFIRS-P is divided by the number of items.

Computerized Test of Variables of Attention (TOVA). Four domains: Response time (RT), variability of response time (VRT), omission errors (OE), and commission errors (CE).
