# Supplementary material for: Outcomes of a 12-week ecologically valid observational study of first treatment with methylphenidate in a representative clinical sample of drug naïve children with ADHD
Source: PLoS One. 2021 Oct 21;16(10):e0253727. doi: 10.1371/journal.pone.0253727 (PMC8530346; doi:10.1371/journal.pone.0253727)
Supplement: S8 Table — (PDF) [file pone.0253727.s009.pdf]

S8 Table. Follow-up of adverse reactions (*n* = 187)

BSSERS-C	Week 0 M (SD)	Week 12 M (SD)	Week 0 versus week 12			
			M dif. (SD)	95% CI	t(df)	*p*-value
Insomnia	1.8 (2.2)	1.9 (1.9)	0.0 (2.4)	(-0.4, 0.3)	-0.3 (186)	0.782
Nightmares	0.5 (1.1)	0.1 (0.5)	0.4 (1.2)	(0.2, 0.5)	4.2 (186)	< 0.001
Staring	0.9 (1.5)	0.5 (1.0)	0.4 (1.3)	(0.3, 0.6)	4.6 (186)	< 0.001
Talks less	0.4 (1.2)	0.4 (0.9)	0.0 (1.0)	(-0.1, 0.2)	0.3 (186)	0.711
Disinterested in others	0.4 (1.1)	0.3 (0.9)	0.1 (1.0)	(-0.1, 0.2)	1.1 (186)	0.257
Reduced appetite	0.7 (1.3)[1]	1.9 (1.5)[1]	-1.2 (1.8)	(-1.5, -1.0)	-9.2 (185)	< 0.001
Irritable	3.4 (2.5)	2.9 (2.3)	0.4 (2.2)	(0.1, 0.8)	2.8 (186)	0.005
Stomachaches	0.5 (1.1)	0.3 (0.8)	0.3 (1.1)	(0.1, 0.4)	3.2 (186)	0.002
Headaches	0.4 (1.1)	0.2 (0.6)	0.3 (1.2)	(0.1, 0.4)	2.9 (186)	0.005
Drowsiness	0.6 (1.0)	0.5 (1.0)	0.2 (1.2)	(0.0, 0.3)	1.9 (186)	0.053
Sadness	1.1 (1.6)	0.7 (1.2)	0.4 (1.8)	(0.2, 0.7)	3.4 (186)	0.001
Prone crying	1.0 (1.7)	0.6 (1.1)	0.4 (1.8)	(0.1, 0.6)	2.9 (186)	0.004
Anxious	0.8 (1.5)[1]	0.7 (1.4)[1]	0.1 (1.2)	(-0.1, 0.3)	1.3 (185)	0.203
Nail biting	3.5 (4.0)	3.0 (3.7)	0.5 (2.5)	(0.1, 0.9)	2. 8 (186)	0.006
Euphoria	1.0 (1.5)	0.4 (0.8)	0.7 (1.4)	(0.5, 0.9)	6.5 (186)	< 0.001
Dizziness	0.0 (0.2)	0.1 (0.3)	0.0 (0.3)	(-0.1, 0.0)	-1.3 (186)	0.202
Tics/nervous movements	0.5 (1.3)[1]	0.4 (1.2)[1]	0.1 (1.3)	(-0.1, -0.3)	1.1 (185)	0.256

Paired t-test between adverse reactions (ARs) of week 0 and week 12. M = mean, M dif. = Mean difference, SD dif. = Standard deviation difference, *n* = number.
Number of participants with observed outcome data: [1]*n* = 186.
Barkley's Stimulant Side Effect Rating Scale, clinician rated (BSSERS-C), 17 items, single item [range 0-9].
Irritable symptoms are measured during the day where methylphenidate did not have effect.
