# Supplementary material for: Outcomes of a 12-week ecologically valid observational study of first treatment with methylphenidate in a representative clinical sample of drug naïve children with ADHD
Source: PLoS One. 2021 Oct 21;16(10):e0253727. doi: 10.1371/journal.pone.0253727 (PMC8530346; doi:10.1371/journal.pone.0253727)
Supplement: S9 Table — (PDF) [file pone.0253727.s010.pdf]

S9 Table. Baseline characteristics and symptoms in week 0 of responder and nonresponder (*n* = 199)

	**Responder *n* = 168 (%)**	**Nonresponder *n* = 31 (%)**	**Responder versus nonresponder**			
			**X^2 (df)**		***p*-value**	
**Boys**	125 (74.4)	25 (80.6)	0.5 (1)		0.459	
**Age (10-12 years)**	59 (35.1)	9 (29.0)	0.4 (1)		0.511	
**Comorbidity ≥ 2 diagnoses**	45 (26.8)	9 (29.0)	0.1 (1)		0.796	
**Cognitive deficits**	44 (26.2)	9 (29.0)	0.1 (1)		0.742	
**Conduct disorder**	17 (10.1)	5 (16.1)	1.0 (1)		0.327	
**Clinician rated symptoms in week 0**	**M (SD)**	**M (SD)**	**M dif. (SD error)**	**95% CI**	**t(df)**	***p*-value**
**Inattention**	19.7 (3.7)	20.8 (3.3)	-1.1 (0.7)	(2.6, 0.3)	-1.5 (197)	0.127
**Hyperactivity-Impulsivity**	17.5 (5.7)	21.5 (4.4)	-4.0 (1.1)	(-6.1, -1.8)	-3.7 (197)	< 0.001
**CGI-S**	5.2 (0.9)	5.9 (0.8)	-0.65 (0.2)	(-1.0, -0.3)	-3.6 (197)	< 0.001
**BSSERS-C**	17.0 (10.4)	21.3 (11.3)	-4.3 (2.1)	(-8.4, -0.2)	-2.1 (197)	0.038
**Reduced appetite**	0.6 (1.2)	1.0 (1.8)	-0.4 (0.2)	(-0.9, 0.1)	-1.6 (197)	0.114

Paired t-test and X^2 (chi-squared) between responder and nonresponder. M = mean. M dif. = Mean difference. SD dif. = Standard deviation difference.
ADHD-Rating Scale, clinician rated (ADHD-RS-C, DuPaul). Inattention subscale: 9 items [range 0-27]. Hyperactivity-Impulsivity subscale: 9 items [range 0-27].
Normalisation (zero standard deviation), borderline normalisation (one standard deviation), no normalisation or borderline normalisation (two standard deviations) of ADHD cores symptoms (ADHD-RS) due to Danish norms of sex and age.
Nonresponder defined by no normalisation or borderline normalisation of any subscales of ADHD-RS-C in week 12 or by discontinuation of treatment due to adverse reactions.
Age between 7 and 9 years old or between 10 and 12 years old. Cognitive deficits: *Inferioritas intellectualis* (DR 41.8) from the diagnostic conference and/or from the intelligence test (WISC) (IQ 70-85) depending on data access or no cognitive deficits (IQ ≤ 86). Conduct disorder: *Hyperkinetic conduct disorder, conduct disorders, mixed disorders of conduct and emotions* (DF 90.1, DF 91.X, DF 92.X) or no conduct disorder.
Clinical Global Impression Severity (CGI-S) week 0: 1 item [range 1-7]. Barkley's Stimulant Side Effects Rating Scale, clinician rated (BSSERS-C). Whole scale (17 items) [range 0-153]. Reduced appetite (single item) [range 0-9].
