# Supplementary material for: Outcomes of a 12-week ecologically valid observational study of first treatment with methylphenidate in a representative clinical sample of drug naïve children with ADHD
Source: PLoS One. 2021 Oct 21;16(10):e0253727. doi: 10.1371/journal.pone.0253727 (PMC8530346; doi:10.1371/journal.pone.0253727)
Supplement: S1 File — (PDF) [file pone.0253727.s011.pdf]

INDICES: INDIvidualised drug therapy based on pharmacogenomics: focus on carboxylesterase 1 (CES1)

1. SUMMARY

To a large extent drug treatment is still based upon the one-size-fits-all principle. This is unfortunate as there are large individual variations in the response to many drugs. Often a significant fraction of patients receiving a drug do not experience any benefit from the agent; others develop severe adverse effects. This project proposes the application of a new strategy that combines pharmacological, genetic and metabolomic sciences to improve pharmacotherapy of important mental and somatic disorders. It focuses upon carboxylesterase 1 (CES1), a key enzyme in the metabolism of a variety of essential drugs, including methylphenidate (MPH) and trandolapril (TA) for treatment of attention-deficit hyperactivity disorder (ADHD) and chronic heart failure (CHF), respectively. The aims are to 1) pioneer clinical decision strategies allowing individualisation of treatments with MPH and TA; 2) develop new research strategies that may serve as paradigms for future studies on individualised drug treatment; 3) identify drugs which interact with CES1 and produce toxic effects when administered together with MPH or TA; and 4) develop fast methods with commercial potential for predicting MPH and TA responses. The study is the first to develop guidelines for individualised treatments with MPH and TA. The approach is unique as it combines genomics, metabolomics and systems pharmacology. Implementation of guidelines for individualised pharmacotherapy is expected to improve treatment efficacies and reduce the risk of adverse effects, leading to reductions in health care system costs and improved patient quality of life. Moreover, the project should provide clues to individualisation of therapies with other essential CES1-dependent drugs (e.g. Tamiflu® and antihypertensive agents related to TA) and inspire future studies of the etiology of lipid metabolism disorders.

2. OBJECTIVES

The vision and overall scientific objective of INDICES is to identify causes of individual variation in drug responses to CES1-dependent drugs among patients with selected psychiatric and somatic disorders and translate this knowledge into clinical tools that can improve treatment efficacy and reduce the risk of adverse effects. The specific objectives are to provide psychiatrists and cardiologists with easy-to-use laboratory analyses and clinical guidelines for individualised treatments of ADHD and CHF with MPH and TA, respectively. These clinical objectives are dependent on a series of basic scientific objectives being met: 1) achievement of detailed knowledge of genetic variants of the key hepatic enzyme, CES1, and the impact of these variants on rate of drug metabolism; 2) identification of lipid metabolites and metabolomic signatures acting as proxies for CES1 activity; and 3) the combination of pharmacological, genetic, metabolomic and clinical information to accurately predict individual drug responses. The main objective related to society and the economy in Denmark is to improve the quality of life of patients with ADHD and CHF, reduce health care system expenditures and commercialise developed laboratory analyses into fast and easy-to-use tests.

3. THE MAIN RESULTS OF THE PROJECT

1) **Identification of biomarkers predicting individual drug metabolism**. We expect to develop a catalogue of CES1 gene variants that are phenotypically characterised in terms of pharmacokinetic impact and clinical relevance. This includes CES1 gene duplication and its effect on drug metabolism, i.e. a potential “gene-dosage effect”. Furthermore, lipid metabolites

and metabolomics signatures acting as surrogate markers of CES1 activity to predict individual responses to MPH and TA will be identified.

2) **Improvement of drug safety and quality of treatment.** We expect to develop guidelines for individualised treatment with MPH and TA. Also clues to individualisation of other essential drugs metabolised by CES1 will be provided. Furthermore, drugs which interact with CES1 resulting in clinically relevant drug-drug interactions will be identified.
3) **Acquisition of knowledge on the basic regulation of the CES1 enzyme.** We expect to gain new insights into the physiological processes in which CES1 is involved, in particular lipid metabolism, and to formulate hypotheses on the development of disorders such as the metabolic syndrome which may potentially result from deregulation of these processes.
4) **Analytical procedures with commercial potential.** Analytical procedures developed by the study will be made into kits. This includes a procedure for determination of gene copy number as well as CES1 genotyping kits serving as companion diagnostics to treatment with MPH and TA.
5) **Paradigm for future studies.** The use of metabolomics in combination with systems pharmacology and genomics may serve as a conceptual paradigm for future studies of individual variation in drug metabolism.

4. BACKGROUND AND HYPOTHESIS OF THE PROJECT

The prevalence of ADHD in children and CHF is rising in Denmark, where these diseases now affect 6-8% of the population (1,2). This is accompanied by increased use of MPH and ACE inhibitors (ACE-Is). For example, the number of ADHD patients treated with MPH has increased by a factor of 10 over the last 10 years in Denmark (3). The use of this drug is expected to increase further due to increased focus on adult ADHD. In 2009 the consumption of MPH and ACE-Is in Denmark was equivalent to treating a total of 25,000 patients with ADHD and 340,000 with CHF (3).

There are large individual variations in the response to commonly used drugs. Often a large percentage of patients do not experience any benefit from the drug they are given; others develop adverse effects. For MPH the non-response rate is in the range of 15-20% (4). Adverse effects during treatment of ADHD with MPH are exceedingly common and include gastrointestinal disorders, insomnia and nervousness with incidences above 5%. Cardiac symptoms are also common with incidences of 3-5%. Discontinuation of MPH treatment due to severe adverse effects occurs in about 1% of cases.

Antihypertensive drug treatment is largely based on a trial and error approach, reflecting a marked individual variation in the response to different drug classes, including the ACE-Is (5,6). Animal studies have suggested that pharmacokinetic factors are involved in the non-response to ACE-Is, implying that dose adjustments can improve treatment efficacies (7). Non-serious adverse drug reactions are common in treatment with ACE-Is, but some patients develop severe and potentially life-threatening adverse effects such as renal impairment. Together with MPH the ACE-Is are on the top 10 list of drugs causing adverse effects in Denmark (3).

CES1 plays a key role in the metabolism of a variety of ester- and amide-containing xenobiotics and endogenous components, including MPH, TA, oseltamivir (Tamiflu®) for combating flu pandemics, the anticancer drug capecitabine, the analgesic meperidine and cocaine (8). Relatively few single nucleotide variations have been identified in CES1 and overall there is a hiatus in the knowledge of variation at the CES1 locus (9). Recent data from a collaborative study supported by the European Commission (EU-FP7) have revealed duplication of the CES1 gene at frequencies above 0.20 in Europe (Henrik Berg Rasmussen, unpublished observations). Gene duplication may give rise to the formation of increased amounts of the gene product, i.e. a “gene dosage effect”.

Genetic screening of drug metabolising enzymes such as CYP2D6 provides the clinician with the ability to identify patients with abnormal rate of drug metabolism and to individualise the treatment based on this. For example, a CYP2D6 “ultra-rapid metaboliser” may not experience any benefit from treatment with a standard dose of a CYP2D6-dependent drug and should have increased doses (10). Therapeutic drug monitoring is an alternative tool to identify patients with abnormal drug metabolism rate. The main drawback is that it cannot be used until after therapy initiation delaying clinical decision making. Moreover, drug monitoring is of limited value for treatment with drugs, such as MPH, for which there is not a plasma concentration range defining an optimal response. There are large individual variations in the metabolism of MPH, TA and oseltamivir, all CES1-substrates (11,12,13), suggesting a significant potential for individualisation of therapies with these drugs. Except for an association of two single nucleotide polymorphisms (SNPs) in CES1 with poor metabolism of MPH that included a steroselective effect on the metabolism of the l-form of this drug (14), associations between *CES1* genotype and drug metabolism have not been reported.
Drug-drug interactions are also a general problem for pharmacotherapy particularly relevant to treatment of ADHD with MPH as this disorder often co-exists with other treatment-requiring mental or somatic disorders. No studies have systematically assessed the risk of toxic effects due to drug-drug interactions during co-medication of MPH with other drugs (15). In this regard systems pharmacology, a new area of pharmacology that uses network analyses to examine drug actions, holds promises for improving understanding of the underlying mechanisms behind a drug’s effect, adverse effects and drug-drug interactions (16).
Besides being involved in the metabolism of essential drugs, CES1 is an important player in endogenous lipid metabolism as it hydrolyses cholesteryl esters and triglycerides, suggesting a role for this enzyme in the regulation of intracellular levels of these substances (17). This combined with findings indicating that CES1 activity is regulated by various lipids (18) hints at a relationship between the level of lipid metabolites in the blood and rate of metabolism of CES1-dependent drugs. A powerful tool to identify novel small molecule markers is metabolomics, which in principal targets the entire repertoire of metabolites within a sample, integrating all environmental influences from gene transcription through protein expression to formation of metabolites (19). Pharmacometabolomics, i.e. the use of metabolomics for examination of drug responses, represents a new and promising tool for individualisation of the drug treatments.

We hypothesise that:

1) Several clinically relevant CES1 variants with an effect on enzyme activity remain to be identified.
2) Duplication of CES1 is associated with increased rate of metabolism of MPH and TA through a “gene dosage effect”.
3) Guidelines for individualised therapy with MPH and TA can be established.
4) Many commonly used drugs interact with CES1, resulting in metabolism-mediated toxic effects.
5) Lipid metabolites are proxies for CES1 activity and correlated with the rate of metabolism of CES1-dependent drugs.
6) The combination of pharmacology genomics and metabolomics which bridges genotype to phenotype is more effective in predicting individual drug responses to CES1-dependent drugs than each of the two “omics” alone and integration of pharmacology with genomics and metabolomics into “pharmacolomics” is the most promising solution to individualised medicine.

5. INNOVATIVE VALUE, IMPACT AND RELEVANCE OF THE PROJECT

Innovations and novel concepts

1) First application of metabolomic profiling using knowledge on endogenous substrates of a drug-metabolising enzyme to examine the basis of individual differences in pharmacokinetics.

2) A complete catalogue of CES1 variants, including duplication with information about the effect on drug metabolism, is highly desired but currently does not exist.
3) Combination of genomics and metabolomics, which accumulates all environmental influences onto the genetic background with systems pharmacology is unique.
4) First to develop guidelines for individualised treatment with MPH and TA.
5) Identification of hitherto unknown drug-drug interactions may improve therapies with MPH, TA and other drugs.
6) An easy and reliable method for determination of gene copy number.

Impact on future research

1) Redirection of the current line of research by a combination of metabolomics with genomics - a conceptual paradigm for future studies of individual drug metabolism and response.
2) Fuelling studies of genetic variability in the metabolism of an entire range of CES1-dependent drugs, including several ACE-Is, oseltamivir (Tamiflu®) and cocaine.
3) Fuelling studies of individual susceptibility of obesity/lipid metabolism disorders.
4) *In-silico* and *in-vitro* identified drug-drug interactions call for *in-vivo* confirmation.

Relevance to society. Individualised treatment with of ADHD and CHF with MPH and TA, respectively, has the potential to improve treatment efficacy and reduce the risk of adverse effects, resulting in improved patient quality of life and in reduced expenditures to the health care system. We expect to develop an assay with a commercial potential and to materialise new variants of CES1 into a kit for a rapid routine genotyping prior to initiation of treatment with CES1-dependent drugs, a companion diagnostic. This may create new jobs related to kit development and manufacturing.

6. PROJECTS METHODOLOGY AND RESULTS

INDICES consists of seven closely interconnected work packages (WPs).

WP1: "Drug and lipid metabolite study". The purposes are to: 1) develop and validate methods for determination of plasma concentrations of TA, the d- and l forms of MPH, and the major metabolites of these drugs; 2) detect *in-vitro* drug-drug interactions of potential clinical relevance; and 3) identify endogenous lipids modulating the activity of CES1. The methods for quantification of the two drugs and their metabolites will be based on liquid chromatography-tandem mass spectrometry. Plasma extractions of MPH and its major metabolite will be performed by a mixed-mode solid-phase extraction procedure followed by chromatography on a chiral column. The developed methods will be applied for analysis of the samples from WP2 and WP7. We will use *in-silico* data generated by WP5 to select commonly used drugs that potentially interact with CES1. The inhibitory effect of these drugs on CES1will be examined by *in-vitro* systems of pooled human liver microsomes or recombinant CES1. This involves co-incubation of the potential inhibitor with one of the two CES1-selective drugs, MPH or TA, followed by determination of the concentrations of the CES1-selective drug. We will also use the *in-vitro* system to screen lipid libraries for identification of lipids interacting with CES1. Furthermore, lipid metabolites WP4 associates with a pharmacokinetic profile will be examined in the *in-vitro* system to verify an effect on CES1 activity. In the event of problems with the development of the assays for detection of MPH, TA and their metabolites, we will have the samples analysed at an academic institution or analytical companies abroad. Furthermore, an alternative approach based on a "coloured" enzyme substrate for detection of drugs and lipids interacting with CES1 will be developed. Overall, we do not expect delay in excess of two months. Such a delay would not be critical.

WP2: "Pharmacokinetic study". The purposes are to: 1) correlate the CES1 genotype and copy number with the pharmacokinetic profile of TA and MPH and to determine dose adjustment factors for these drugs; 2) provide pharmacokinetically well-characterised DNA samples for the WP 3; and 3) provide data for WP 4, 6 and 7. We will recruit 100 healthy, young adults at the Department of

Science, Systems and Models, Roskilde University, where we have permission to contact the students. After screening the recruited subjects for CES1 gene copy number, we will select 5 CES1-duplication homozygotes, 10 duplication heterozygotes, and 20 subjects' homozygous without duplication. This sample of subjects enriched for the CES1 gene duplication will participate in two pharmacokinetic trials with MPH and TA, respectively, separated by a "wash-out" period. The trials will take place at the experimental pharmacological phase 1 unit, Department of Clinical Pharmacology, Bispebjerg University Hospital. After pre-drug blood sampling and intake of a single dose of one of the two drugs in the morning, there will be blood sampling throughout the rest of the day. We will correlate the pharmacokinetic profile with the CES1 genotype and estimate dose adjustment factors for genotypes conferring decreased or increased metabolism of TA and MPH. Using *in-silico* modelling we will identify other CES1-dependent drugs and evaluate their potential as candidates for individualised therapy. Successful outcome of WP2 depends upon the ability to recruit the desired number of subjects and avoid drop-out of recruited subjects. We will solve these problems by 1) motivating oral presentations, 2) a relatively high economic compensation to each study subject, e.g. DKK 3,000-3,500 (EUR 350 Euros) per day, 3) additional recruitment at the University of Copenhagen, and 4) use of the homepage, www.forsoegsperson.dk, which is dedicated to recruitment of subjects to biomedical studies. A major drop-out of participants after the first pharmacokinetic study may necessitate a new recruitment round. This may cause a two to three-month delay, which is not critical.

WP3: "Genetic study". The purposes are to: 1) identify new variants in the CES1 gene and in its proximity; 2) assess their functional significance; 3) establish analyses for genotyping of clinically relevant CES1 variants; 4) assess a relation between lipid disorders and CES1; and 5) determine the worldwide frequency of CES1 duplication and identify nutritional factors acting in a positive selection. As the first step we will examine the samples from the 100 young adults recruited by WP2 by using paired end-mapping and Solexa sequencing of a 1.5 Mb segment containing CES1. The functional importance of identified variants will be assessed using the pharmacokinetic data from WP2 supplemented with *in-silico* analyses. Based on this we will construct a catalogue of CES1 variants and design genotyping assays for those of potential clinical relevance. SNP genotyping will be based on the 5'exonuclease-based approach. A novel high-throughput method for determination of gene copy number will be developed. The MPH treatment response group will be used as a discovery cohort and IP protected. In collaboration with deCODE, sequence variants associated with MPH treatment response will be imputed into a large ADHD sample (N>1000) and tested for association with ADHD. Likewise, a search for genetic associations of CES1 with lipid metabolism disorders will be conducted in collaboration with deCODE. We will also determine the worldwide frequencies of CES1 duplication and correlate them with the amount of meat and dairy products in the diet as proxies for the levels of triglycerides and cholesterol. In the event of problems with the Solexa-based sequencing, other technical platforms will be applied. The delay resulting from this should not exceed four months, which is not critical.

WP4: "Pharmacometabolomics". The purposes are to: 1) identify lipid metabolites and pathways implicated in variation in MPH and TA metabolism; and 2) improve prediction of individual variation by combination of metabolomic profiles and DNA markers. We will determine metabolomic profiles of plasma samples from all of the participants in the pharmacokinetic study prior to intake of MPH and TA and determine predose-metabolomic profiles. As the participants in the first pharmacokinetic trial (MPH) are identical to those in the second pharmacokinetic trial (TA), we will obtain two independent pre-dose profiles of each participant. Choice of a metabolomic profiling platform will be based upon knowledge on endogenous substrates of CES1 employing cholesterol and triglyceride biochemistry analysis platforms, each targeting about 5,000 different lipid metabolites in an integrative approach with subsequent partial least square modelling

(PLS) of data. The latter is a multivariate statistical approach, which will detect relationships between pharmacokinetic parameters and lipid metabolites, thus allowing us to build a statistical model capable of predicting individual variations in the rate of drug metabolism. Subsequently we will incorporate the data from deep sequencing of the CES1 gene and copy number determinations, into the PLS analyses and remodel to increase the pharmacokinetic prediction ability. To provide insights into the background of individual variability in pharmacokinetics we will construct hypothetical metabolic networks based upon metabolites associated with individual variability in WP2 and their interacting partners using information from the Human Metabolome Database. The information contained in each of these metabolomics networks will be condensed into a single, clinically applicable measure. In the event of problems we will use our extended network which includes the Pharmacometabolomics Center, Duke University to solve the problems.
WP5: "Systems pharmacology and metabolomics". The purposes are to: 1) identify drug-drug interactions of potential importance to the metabolism of CES1 dependent drugs by *in-silico* methods; 2) identify genetic factors associated with adverse effects of MPH and TA; 3) provide a deeper understanding of the basis of individual variation in CES1-mediated drug metabolism; and 4) provide essential bioformatics services to WP1, WP2 and WP3. We will collect information on structural parameters of drugs and drug-like compounds interacting with CES1. Based on this we will construct *in-silico* models for virtual screening of large compound databases (millions of compounds) to detect drugs potentially involved in interactions with CES1-dependent drugs. A systems pharmacology network will be developed by the compilation of drug-target interactions and protein-protein interactions of the targets. Information on adverse effects will be integrated into this network to identify genes and complexes of genes implicated in individual susceptibility to adverse effects of MPH and TA. Using the phenome-interactome platform in combination with the Human Metabolome Database, genes encoding proteins that directly or indirectly interact with CES1 will be identified and mapped to genetic variations obtained from WP3 and the literature. This will allow us to create a platform for understanding individual CES-1 mediated drug metabolism and to develop an *in-silico* model for prediction of individual variation in the metabolism and response of CES1-dependent drugs. The Center for Biological Sequence Analysis is in close contact with other leading bioinformatics clusters. In the event of problems one or more of these will be contacted.
WP6: "Clinical study A - ADHD". The purposes are to: 1) examine the relationship of CES1 genotype with the response to MPH in children with ADHD, and 2) develop guidelines for CES1 genotype-based prescription of MPH for treatment of ADHD in children. We will recruit 200 drug-naïve children aged 7-11 years with ADHD according to ICD-10 criteria. Rating of ADHD symptom severity and adverse effect rating will be conducted at base-line. MPH will be initiated at a low dose and increased until symptom normalisation or treatment failure occurs. CES1 genotypes including copy number will be correlated with the drug response measures: 1) number of weeks required for a predetermined improvement to occur, 2) occurrence of treatment failure, and 3) adverse effects and premature discontinuation of therapy due to unacceptable adverse effects. For example, this will allow us to assess whether the MPH dose should be increased at a faster rate and with larger dose increments than normal in patients with CES1 duplication. We also expect to determine whether patients with defective alleles should be up-titrated using smaller dose increments than normal. By combining the pharmacokinetic data from WP2 we will develop guidelines for individualised therapy of ADHD with MPH. To enhance recruitment participating children will be offered a DKK 50 (EUR 7) gift certificate at a toy shop. In the event of recruitment delay we will also recruit at *Privathospitalet, Hejmdal* with permission from its chief psychiatrist, Dr Torsten Warrer. Professor Hans-Christoph Steinhausen, Aalborg Psychiatric Hospital, will also assist in the recruitment if necessary. Determination of plasma concentrations of MPH has limited therapeutic value and will not be done.

WP7: "Clinical study B – CHF". The purposes are to: 1) examine a relationship of CES1 genotypes with the response to TA in patients with CHF and 2) develop guidelines for genotype-based prescription of TA for treatment of CHF. A similar prospective design as employed in WP6 will be used. A total of 200 patients with CHF, left ventricular ejection fraction <0.45, and clinical indication for TA will be recruited. TA is initiated followed by successive dose-titration to a predetermined target dose with drug discontinuation or dose reduction if adverse effects occur. The investigation duration is 6 months with recruitment of patients at the out-patient clinics at two large cardiology hospital departments in Copenhagen (Gentofte University Hospital and Bispebjerg University Hospital). If necessary, supplementary recruitment will be possible at the Department of Cardiology B, Rigshospitalet and the Department of Cardiology, Hillerød University Hospital. Patients will be clinically examined at t=0, 3 and 6 months and blood and urine samples taken. CES1 genotype will be correlated to clinical and biochemical treatment response measures. In combination with the results from WP2, and drug metabolite measurements in plasma from the CHF patients, guidelines for individualised treatment of CHF patients with TA will be developed. They may include dose adjustments for 'poor metabolisers' and 'ultrarapid metabolisers' or recommendation to use drugs which are not metabolised by CES1 for treatment of patients with abnormal CES1 genotypes. During a PhD student study visit at Newcastle University, the development of the treatment guidelines will take place in collaboration with Simon Thomas, Professor of Clinical Pharmacology and Therapeutics.
Power calculations. Calculations for the pharmacokinetic trial with the ratio between minimum difference in mean area under curve and SD = 3, $\alpha = 0.05$, gives power = 0.94 with 35 participants. For the clinical trials we assume a smaller effect size, namely, 1.5 and for $\alpha = 0.05$, this gives power = 0.90 with 200 participants. Therefore all of the trials are very well powered.

7. PROJECT PLAN

The Gantt diagram only includes scientific personnel. Light brown denotes PhD student and postdoc study visits.
WP1 (Project leader: Kristian Linnet). A PhD associated with WP1 will execute the following activities within the time frame indicated below:

- Month 1-12: development of analyses for quantification of drug and drug metabolites
- Month 12-15: analysis of samples from pharmacokinetic study
- Month 15-21: study visit (Linkobing University)
- Month 21-24: analysis of samples from the CHF study
- Month 24-30: identification of drugs interacting with CES1
- Month 30-36: identification of bioactive lipids interacting with CES1
- Month 36-39: preparation of PhD dissertation

We expect the drug-drug interactions analyses and analyses of bioactive lipids to start after 12 months. This should allow enough time for their completion after 30 months. Kristian Linnet will contribute with 3 months of work for execution of WP1. Olivier Taboureau will contribute with 2 months, Henrik Rasmussen with 2 months, Kim Dalhoff or Gesche Jürgens with 1 month of work. Kristian Linnet will provide 3 months of technical laboratory assistance.
WP2 (Project leader: Kim Dalhoff). A PhD associated with WP2 will execute the following activities within the time frame indicated below:

- Month 1-9: planning and execution of pharmcokinetic trials
- Month 9-15: study visit (Kiel University)

DICES - Gannt diagram

Year	2011				2012				2013				2014				2015				Months							
**Projects / quarter**	**Q1**	**Q2**	**Q3**	**Q4**	**Q1**	**Q2**	**Q3**	**Q4**	**Q1**	**Q2**	**Q3**	**Q4**	**Q1**	**Q2**	**Q3**	**Q4**	**Q1**	**Q2**	**Q3**	**Q4**	**Sct. Hans**	**UniCph**	**BBH Pharma**	**de CODE**	**Leiden**	**CBS**	**GLO Psyc**	**GEH Card**
**Work Package 1**																												
Drug metabolite detection																					2	3+1phd	1			2		1
**Work Package 2**																												
Pharmacokinetic study																					4	1	6+1phd			1		
**Work Package 3**																												
Genetic study																					6+1postdoc			3		1		
**Work Package 4**																												
Pharmacometabolomics																					1		1		2+2 years	1		
**Work Package 5**																												
Systems pharmacology																					2		1			4+1phd		
**Work Package 6**																												
Clinical study A																					2		1				4+1phd	
**Work package 7**																												
Clinical study B																					1	1	1					4+1phd

Thomas Hankemeier’s staff at the Netherlands Metabolomics Centre will contribute 2 years of work. Thomas Hankemeier will use 2 months on WP4.

- Month 15-21: calculation of pharmacokinetic parameters and analyses of their relationship with CES1 genotype
- Month 21-27: guidelines for genotype-based adjustment of doses of MPH and TA
- Month 27-39: identification of additional candidates for genotype-based adjustment of doses among other CES1 dependent drugs
- Month 39-42: preparation of PhD dissertation

The pharmacokinetic trials are labour-intensive, requiring the study subjects to be split into smaller groups participating on separate days. Therefore a relatively large amount of time has been allocated to the completion of these trials. The study visit is placed in the period where the samples from the pharmacokinetic trials are being analysed, allowing for the best use of the time. Kim Dalhoff and Gesche Jürgens will contribute with 6 months of work and Henrik Rasmussen with 4 months of work for completion of WP2. Olivier Taboureau will use 1 month of work onWP2 and Kristian Linnet, or his PhD student, with 1 month of work. A laboratory technician (self-financed, MHC Sct. Hans) will contribute with 2 months work and be responsible for registering samples, extraction of DNA and CES1 gene copy number determination. In addition it will be necessary to have two nurses contribute 1½ month of work at the experimental pharmacological unit.

WP3 (Project leader: Hreinn Stefansson). A postdoc associated with WP3 will execute the following activities within the time frame indicated below:

- Month 1-3: introduction and planning
- Month 3-9: deep sequencing of the 100 samples (Solexa sequencing)
- Month 9-15: elaboration of a comprehensive catalogue of genetic variants of CES1 and variants in neighbour genes, construction of haplotypes and *in-silico* assessment of the functional importance of the variants
- Month 15-27: development of genotyping assays
- Month 27-33: genotyping of the clinical samples
- Month 33-39: determination of CES1 duplication in different populations of the world and evolutionary analyses
- Month 39-42: preparation of manuscripts and finalisation of the activities

After the introductory period the postdoc will then take residence at deCODE for one year. Hreinn Stefansson is expected to contribute to WP3 with 3 months of work, Henrik Rasmussen with 6 months of work and Olivier Taboureau with 1 month. MHC Sct. Hans will provide 6 months of technical laboratory assistance (self-financed).

WP4 (Project leader: Thomas Hankemeier). The activities in WP4 will take place at the Netherlands Metabolomics Centre and the Metabolomics Center, Duke University. The following activities will be executed within the time frame indicated below:

- Month 1-12: metabolomics analyses of plasma samples
- Month 12-24: building of statistical models for prediction of individual variations in the rate of metabolism of MPH and TA
- Month 24-30: analysis of combined pharmacometabolomics and genetic data
- Month 30-39: construction of hypothetical metabolomic networks based upon metabolites associated with individual variability in pharmacokinetic study.

Thomas Hankemeier will conduct the metabolomics analyses of the plasma samples. Modeling of metabolomics data, pathway analyses and construction of hypothetical metabolomic networks will take place at the Netherlands Metabolomics Centre or the Pharmacometabolomics Center, headed by Rima Kaddurah-Daouk who will collaborate on the project. The PhD associated with WP5 is expected to complete a 3-month study visit at the Netherlands Metabolomics Centre or the Pharmacometabolomics Center. Thomas Hankemeier will contribute to the completion of WP4 with

2 months of work, Olivier Taboureau with 1 month and Kim Dalhoff or Gesche Jürgens with 1 month of work. Henrik Rasmussen will contribute with 1 working month.

WP5 (Project leader: Søren Brunak). A PhD associated with WP5 will execute the following activities within the time frame indicated below:

- Month 0-9: identification of drugs interacting with CES1
- Month 9-15 months: identification of genes involved in adverse effects of MPH and TA
- Month 15-18: study visit at the Netherlands Metabolomics Centre or the Pharmacometabolomics Center, Duke University
- Month 18-21: combining systems pharmacology data and pharmacometabolomic data
- Month 21-33: development of *in-silico* tool for prediction of individual response to CES1-dependent drugs
- Month 33-36: preparation of PhD dissertation

Olivier Taboureau will contribute to the completion of WP5 with 4 months, Henrik Rasmussen with 2 months, and Kim Dalhoff or Gesche Jürgens with 1 month of work.

WP6 (Project leader: Tine Houmann) and WP7 (Project leader: Peter Riis Hansen). A PhD associated with WP6 and another with WP7 will execute the following activities within the time frame indicated below:

- Month 0-3: planning and introduction
- Month 3-15: recruitment of 100 patients who will then complete a 6-month study period
- Month 15-27: recruitment of 100 additional patients who will then complete a 6-month study period corresponding to a total of 200
- Month 27-33: study visit at University of Nijmegen (WP6) and University of Newcastle (WP7)
- Month 33-39: statistical treatment of data and elaboration of manuscripts
- Month 29-42: preparation of PhD thesis

Tine Houmann (WP6) and Peter Riis Hansen (WP7) will both contribute with 4 months of work, Henrik Rasmussen with 2 months on WP6 and 1 month on WP7. Kim Dalhoff and Gesche Jürgens will contribute jointly with 1 month of work for completion of WP 6 and 1 month for completion of WP7. Kristian Linnet will contribute with 1 month of work to WP7. Henrik Rasmussen will provide 3 months of technical laboratory assistance (self-financed).

8. PROJECT'S INTERNATIONAL DIMENSION

Contact and exchange of knowledge with the international science community will be enhanced by:

1) The participation of leading research institutions from abroad in the project
2) PhD student study visits at research institutions abroad for a period of 3-6 months. Agreements have been obtained from the Department of Forensic Genetics and Toxicology in Linkobing, University of Nijmegen, Kiel University, Duke University and Newcastle University
3) Attendance by all of the PhD students and key persons in at least one, preferably two, international high-level scientific congresses annually.
4) Annual work meetings (cf. "11. The Participating parties and project management").
5) Mid-term evaluation (cf. "11. The Participating parties and project management")

The interest in personalised medicine has a high priority globally. The concept of combining metabolomics and genomics to provide insights into the basis for individual variation of drug responses is in line with that of a recent NIH-funded initiative, "National Metabolomics Network for Drug Response Phenotypes". The current application is connected to this network through the Netherlands Metabolomics Centre which is participant of this network. We expect the international activities to have a major impact on the future research of individualised medicine in Denmark by: 1) enhancing contact with leading scientists within the field and facilitating the exchange of ideas

and future collaborations, 2) offering opportunities for the scientific education of young scientists at the highest level, and 3) importing knowledge that Denmark currently does not possess, particularly in regard to pharmacometabolomics. Currently, most research groups in individualized medicine in Denmark focus upon pharmacogenomics. The international activities of INDICES will serve to redirect this research towards a line that combines several "omics". A combination of the two "extremes", namely genomics and metabolomics that accumulate all of the environmental influences onto the genetic background, could become the most rewarding avenue of research.

9. LEGAL AND ETHICAL ASPECTS

The local Committees on Biomedical Research Ethics and the Danish Data Protection Agency have issued permissions for the clinical WP on ADHD that allow for the collection, management and examination of data as well as the taking of biological samples from psychiatric patients below 18 years of age for research purposes. This includes genetic research projects.

Permissions from the local Committees on Biomedical Research Ethics and the Danish Data Protection Agency are also required for completion of WP2, WP3 and WP7. Subjects will be recruited after having given informed consent. They will be adequately informed about potential risks associated with the studies.

We consider the risks to the subjects participating in the pharmacokinetic study as low since the study subjects will all be healthy young adults with no history of heart disease or other diseases that might increase the risk of severe adverse effects. Moreover, the drugs applied in this study, MPH and TA, are each given as a single, relatively low dose. The conduction of the pharmacokinetic study in an experimental pharmacological phase 1 unit with medical doctors and trained nurses being present during the entire trial also serves to increase the safety of the study subjects. We do not anticipate the clinical studies to be associated with safety concerns since dose adjustments are not implemented at this stage of our research.

10. PUBLICATION AND PROMOTIONAL STRATEGY AND EXPLOITATION OF RESULTS

We expect the quality of our studies to readily meet the publication standards of high-ranked peer-reviewed scientific journals such as *Nature Genetics, PLOS Genetics, The New England Journal of Medicine, Journal of Pharmacology and Experimental Therapeutics, and The Pharmacogenomics Journal, Bioinformatics, Journal of Child and Adolescent Psychopharmacology, Molecular Psychiatry,* and *Cardiovascular Research*. Information about published studies will be deposited in the Danish National Research Database, a central portal for published Danish research. Here we list the topics of a number of publications that we expect to publish: **WP 1**: 1) quantification of MPH and TA, 2) CES1-mediated drug interactions, 3) lipid interactions with CES1, and 4) review of CES1 interactions. **WP2**: 1) CES1 genotype and drug metabolism, 2) dose-adjustment based upon CES1 genotype, 3) individualised drug therapy with CES1-dependent drugs, 4) CES1 variants in relation to drug metabolism. **WP3**: 1) organisation of the CES1 and CES4 loci, 2) methods for identification of CES1 variants, 3) environment, diet and CES1 gene copy number, 4) CES1 gene and lipid metabolism disorders, and 5) method for gene copy number assessment. **WP4**: 1) metabolomics and individual pharmacokinetic profiles, 2) metabolomics profiling of drug metabolising enzymes, and 3) metabolomic network of CES1 interacting lipids. **WP5**: 1) CES1 metabolism-mediated drug-drug interactions, 2) genetic susceptibility to drug adverse effects, 3) *in-silico* tool for prediction of individual drug responses. **WP6**: 1) CES1 genotype and treatment of ADHD with MPH, 2) metabolomic signatures and ADHD treatment, 3) guidelines for CES1 genotype-based therapy with MPH, and 4) CES1 genotype and MPH treatment responses. **WP7**: 1) CES1 genotype and efficacy

of TA, 2) CES1 genotype and lipid markers in heart failure, 3) guidelines for CES1 genotype-based therapy with TA, and 4) metabolomic signatures and prediction of outcome of treatment with TA. Results from INDICES will also be presented at international congresses, e.g. the World Congress on Psychiatric Genetics, the European Society of Child and Adolescent Psychiatry, the International Society of Metabolomics, and the annual meeting of the European Heart Society. Moreover we will prioritise giving presentations at national scientific meetings, e.g. the annual meeting of the Danish Psychiatric Society and to disseminate knowledge at post-graduate courses for medical doctors and through student courses. We will seek permission from the ADHD Association and the Danish Heart Association to inform about INDICES on their homepages as well as suggest presentations of the project to be held at meetings arranged by these patient associations. Since "tailored medicine" has a broad public appeal we will contact large Danish newspapers and national television channels to present the project's main findings to provide inspiration for thematic articles and broadcasts. The implementation of guidelines for CES1 genotype-based prescription of MPH and TA will start at the departments responsible for the clinical studies (Psychiatric Centre Glostrup and Department of Cardiology, Gentofte University Hospital); the Mental Health Centre Sct. Hans will conduct the genotyping. After implementation at these departments we will identify another two hospital ADHD departments and hospital cardiology departments that are interested in introducing the new treatment principles. We will assist these departments with implementation of the guidelines for individualised treatment. We expect that this combined with other efforts such as presentations at national scientific meetings and lectures at post-graduate courses for medical doctors gradually will lead to the implementation of individualised guidelines at a larger number of hospitals in Denmark. We expect to develop assays that can be transformed into easy-to-use laboratory kits for clinical use. The development and marketing of such kits would be a task for a biotechnological company. The technology transfer unit of the capital region (RegionH), Tectra, will assist with patenting and commercialisation of innovations and hold responsibility for the process of commercialisation. Intellectual property rights (IPRs) and collaboration with project participants not belonging to RegionH will be regulated by collaborative agreements written by Tectra before the project starts. Issues on which several parties might claim IPRs and foreground rights will be discussed and agreed upon with Tectra and partners prior to the initiation of the project.

11. THE PARTICIPATING PARTIES AND PROJECT MANGEMENT

The project will be headed by *HBR*. He will be assisted by a management group consisting of *KL*, *KD*, *HS*, *THH*, *SB*, *TH*, and *PRH*. An administrator (0,2 FTE) will be responsible for routine mail, budgets, coordination of meetings and assistance in preparing reports; see organisation chart.

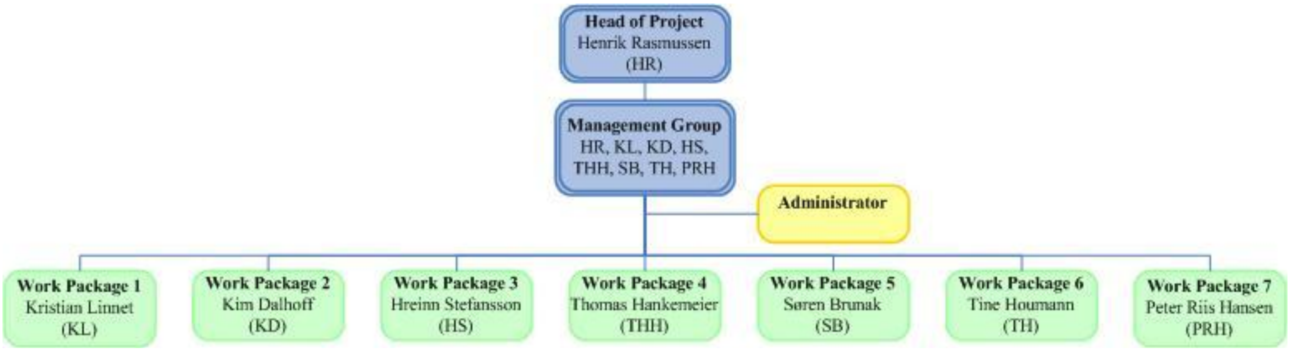

Henrik Berg Rasmussen (HBR), DVM, PhD, is a senior scientist and laboratory manager at the Research Institute of Biological Psychiatry and the Mental Health Centre Sct. Hans. He heads a research group focusing upon pharmacogenetics and individualised medicine. *HBR* is responsible

for the development and implementation of new genotyping techniques and the maintenance of the Danish Psychiatric Biobank. Furthermore, he holds responsibility for the CYP2D6 and CYP2C19 genotyping of clinical samples at the Mental Health Centre Sct. Hans, a service provided to hospitals and general practitioners in Denmark, and for quality control of these analyses through collaboration with international partners. *HBR* is the primary investigator of the ADME (structural variation in genes associated with absorption, distribution, metabolism and excretion of drugs) project, a sub-project of PSYCH GENE (EU-FP7) and of a study on the genetic variability of the dopamine transporter together with four international partners. *HBR* will be heading the consortium. Accordingly, he will be involved in overall coordination of the research efforts of the single WPs, maximising the synergies between the participants. He will also actively take part in the genetic WP (planning and conduction of pharmacokinetic trials, supervision of PhD student, development and design of genetic assays and analyses of samples from the clinical WPs) and the two clinical WPs (development of guidelines for genotype-based treatment with MPH and TA).

Kristian Linnet (KL), MD, DMSc, is a professor of forensic chemistry and the director of the Section of Forensic Chemistry, University of Copenhagen. This unit consists of about 50 employees, including scientific and technical staff. *KL* heads a research group currently including 4 PhD students focusing upon the development of new analytical methods, drug metabolism and pharmacogenetics. *KL* participates in several international collaborations and is advisor of the Clinical and Laboratory Standards Institute and a regional representative of the International Association of Forensic Toxicologists. The Section of Forensic Chemistry, University of Copenhagen, is highly experienced in analysis of psychoactive and cardiovascular drugs in human samples. *KL* will be responsible for the drug and lipid metabolite WP. *KL* will also take part in and interact with the pharmacokinetic WP (provision of drug metabolite data), the pharmacometabolomics WP (exchange of data) and the CHF WP (provision of drug metabolite data).

Kim Dalhoff (KD), MD, DMSc, consultant specialist in Clinical Pharmacology and Hepatology, Bispebjerg University Hospital and associate professor at the Medical School, University of Copenhagen. He is the head of the Danish Poison Control Centre and supervisor of several PhD students investigating the influence of genes on drug metabolism and toxicity. He has been involved in several clinical pharmacokinetic studies, maintains a large international network and was the PI of a large GCP study with a new drug against liver cancer. *KD* will be the primary person responsible for the pharmacokinetic study (WP2). He will also be involved in other WPs, in particular the drug metabolite WP (contributing with expertise on drug-drug interactions), the metabolomics WP (provision of pharmacokinetic data) and the two clinical WPs (collaborating in the development of guidelines for genotype-based treatment with MPH and TA).

Gesche Jürgens (GJ), MD, PhD is a clinical pharmacologist. She is employed as specialist registrar at the Department of Clinical Pharmacology (Bispebjerg University Hospital) and the Danish Poison Control Centre. *GJ* is experienced in the planning and conduction of clinical and pharmacokinetic studies and has worked as principle and co-investigator in research projects dealing with pharmacokinetics, drug metabolism and toxicology. *GJ* will provide assistance to WP2, WP4, WP5, WP6 and WP7.

Hreinn Stefansson (HS) PhD, head of the CNS division deCODE genetics, Reykjavík, Iceland. This company is a global leader in human genetics with expertise in generating, analysing and managing large data sets. *HS* has extended experience in coordinating international research projects through his role as the PI of several collaborative ventures, including SGENE (EU-FP6) and PSYCH GENE (EU-FP7). His fields of expertise fall within in genomics, population genetics and identification of disease genes. *HS* will be responsible for WP3. Moreover he will provide services to other WPs and

synergise with them, in particular the pharmacological WP (provision of genetic data and conduction of statistical analyses) and the metabolomics WP (provision of genetic data).
Thomas Hankemeier (*THH*), MSc, PhD, director of the Netherlands Metabolomics Centre, full professor of analytical biosciences, Leiden/Amsterdam Center for Drug Research, Leiden University. *THH* is the PI of several large research projects on the use of metabolomics for discovery of biomarkers including a project with a budget of 53 M Euro to identify clinically predictive biomarker fingerprints for early disease diagnosis. *THH* will be responsible for the metabolomics WP. He will interact and synergise with the drug and lipid metabolite WP (provision of lipid metabolite data), the pharmacokinetic WP (acquisition of pharmacokinetic data) and the genetic WP (acquisition of genetic data). The Netherlands Metabolomics Centre is a member of a network headed by the Pharmacometabolomics Center, Duke University Medical Center, Durham, USA, which is the home of a multi-institutional interdisciplinary research consortium funded by the NIH and consists of centres of excellence in metabolomics and metabolomic bioinformatics, together with centres for molecular pharmacologic and pharmacogenomic science. Director of the Pharmacometabolomics Center is Rima Kaddurah-Daouk. One of the pioneers of metabolomics and a leading player in this field, Rima Kaddurah-Daouk has built programmes that bridge genetic and biochemical global -omics approaches to bring a deeper understanding of the pathways implicated in disease and in drug response. We are connected to the Pharmacometabolomics Center, through the Netherlands Metabolomics Center. Rima Kaddurah-Daouk and the Pharmacometabolomics Center will be participating in the project.
Søren Brunak (SB), physicist, PhD, DSc, professor and director, of the Center for Biological Sequence Analysis, Technical University of Denmark, one of the largest academic bioinformatics clusters in Europe with more than 100 employees. *SB* is also professor of disease systems biology, The Novo Nordisk Foundation Center for Protein Research, University of Copenhagen and primary investigator of several international research projects. *SB* will be the principal investigator of WP5.
Olivier Taboureau (OT), MSc, PhD, associate professor at the Center for Biological Sequence Analysis, Technical University of Denmark. *OT* has expertise in the integration of large-scale chemical and biological data to examine the interplay between small molecules and systems biology. He has been involved in the development of *in-silico* chemical biology including QSAR systems for toxicity prediction and chemical toxicology networks. *OT* has participated in several EU projects, i.e. Psychgene, DEER, Etox and Danish programme (SMVs) and the Innovative Medicines Initiative (IMI) EU programme. He will be responsible for the bioinformatics in WP1 and WP2 as well as WP5. He will also have responsibility for the exchange of knowledge and cooperation with the Pharmacometabolomics Center at Duke University and its outreach into Europe, e.g. the Netherlands Metabolomics Centre.
Tine Houmann (TH), MD, associate professor in child and adolescent psychiatry at the Medical School, University of Copenhagen and senior hospital physician at Glostrup University Hospital. *TH* is the head of the in-patient unit for preschool children and the ADHD Clinic. She is conducting a preschool ADHD study, and is a co-investigator in the Copenhagen Child Cohort, CCC 2000 Study, from which she has experience in the daily clinical management of scientific studies. *TH* is teaching pre- and postgraduate courses in ADHD, PDD and psychopharmacology, and is a pre- and postgraduate scientific supervisor of medical students and trainees. *TH* will be responsible for the ADHD WP. She will also synergise and interact with the pharmacokinetic WP (acquisition of data and development of guidelines for genotype-based dose adjustments) and the genetic WP (receiving results from genotyping of patients).
Anne Mette Skovgaard (AMS) MD, DMSc, associate professor, chief consultant and head of the research unit at Child- and Adolescent Psychiatric Centre Glostrup, University Hospital of Copenhagen. *AMS* is head of the Copenhagen Child Cohort, CCC 2000 and heads a research group

of three PhD students and three postdocs. She is head of the Infant Mental Health Screening and Intervention Study and the Infant-toddler Clinical Database of the Capital Region as well as supervisor for four PhD students in the field of developmental psychopathology. *AMS* will supervise the WP4 PhD student jointly with *TH*.
Peter Riis Hansen (PRH), DMSc, PhD and associate professor at the Medical School, University of Copenhagen is an invasive cardiologist and consultant at the Department of Cardiology P, Gentofte University Hospital. *PRH* is involved in various areas of cardiovascular research and currently supervises 6 PhD students. *PRH* will be the primary person responsible for WP7. He will also synergise and interact with the pharmacological WP (acquisition of data and development of guidelines for genotype-based dose adjustments) and the genetic WP (receiving results from genotyping of patients).

Project management
The following means will serve to coordinate INDICES and create synergy between the participating parties:
1) Regular contact between the members of the management group through email and telephone, including conference calls.
2) Regular quarterly meetings will take place with key individuals in order to coordinate the overall objectives of INDICES. For those WPs particularly dependent upon each other there will be additional meetings with key people and younger scientist for exchange of results and ideas.
3) Three working meetings during the project period with presentations by the key investigators and their PhD students. These meetings will be held to up-date project participants on the activities in the different WPs and create synergy between participants from different WPs. The working meeting will include key scientists from international research institutions offering study visits for the project's PhD students. This will serve to promote the collaboration and active involvement of external partners in the project and provide opportunities to discuss the study visits and the project as a whole. Working meetings will last 1½ days each.
4) Mid-term evaluation with an advisory panel consisting of two or three internationally recognised specialists. These specialists will in due time receive a report of the achieved results and remaining goals before arriving in Copenhagen, where the evaluation will take place.

In the event of project mismanagement or lack of ability of a participant to achieve the desired milestones within the predetermined period the project leader is authorized to discontinue the grant of this participant and seek for a replacement.
The head of each WP is responsible for developing and carrying out education activities for the PhD associated with the WP in question. For WP2 (pharmacokinetic study) and WP6 (clinical study A – ADHD) the key persons *KD* and *TH* will be assisted by *GJ* and *AMS*, respectively. Contact and collaboration with national and international research groups and networks will primarily be mediated and promoted by the persons heading the individual WP.

12. KEY REFERENCES

1) Petersen DJ, Bilenberg N, Hoerder K, Gillberg C. The population prevalence of child psychiatric disorders in Danish 8- to 9-year-old children. Eur Child Adolesc Psychiatry. 2006:15:71-78.
2) O Nielsen, J Hilden, C Larsen, and J Hansen. Cross sectional study estimating prevalence of heart failure and left ventricular systolic dysfunction in community patients at risk. Heart. 2001: 86: 172–178.

3) Danish Medicines Agency’s homepage: http://www.laegemiddelstyrelsen.dk
4) Dopheide JA, Pliszka SR. Attention-deficit-hyperactivity disorder: an update. Pharmacotherapy. 2009: 29:656-679.
5) Webb AJ, Fischer U, Mehta Z, Rothwell PM. Effects of antihypertensive-drug class on interindividual variation in blood pressure and risk of stroke: a systematic review and meta-analysis. Lancet 2010:375:906-915.
6) Dickerson JE, Hingorani AD, Ashby MJ, et al. Optimisation of antihypertensive treatment by crossover rotation of four major classes. Lancet 1999:12:2008-2013.
7) Windt WA, van Dokkum RP, Kluppel CA, et al. Therapeutic resistance to angiotensin converting enzyme (ACE) inhibition is related to pharmacodynamic and -kinetic factors in 5/6 nephrectomized rats. Eur J Pharmacol. 2008:580:231-240
8) Hosokawa M. Structure and catalytic properties of carboxylesterase isozymes involved in metabolic activation of prodrugs. Molecules 2008:13:412-431
9) Marsh S, Xiao M, Yu J, et al. Pharmacogenomic assessment of carboxylesterases 1 and 2. Genomics 2004:84:661-668.
10) Kirchheiner J, Rodriguez-Antona C. Cytochrome P450 2D6 genotyping: potential role in improving treatment outcomes in psychiatric disorders. CNS Drugs 2009:23:181-191.
11) Haessler F, Tracik F, Dietrich H, et al. A pharmacokinetic study of two modified-release methylphenidate formulations under different food conditions in healthy volunteers. Int J Clin Pharmacol Ther 2008:46:466-476.
12) Zhu HJ, Appel DI, Johnson JA. Role of carboxylesterase 1 and impact of natural genetic variants on the hydrolysis of trandolapril. Biochem Pharmacol 2009:77:1266-1272
13) Wattanagoon Y, Stepniewska K, Lindegårdh N, et al. Pharmacokinetics of high-dose oseltamivir in healthy volunteers. Antimicrob Agents Chemother 2009:53:945-952.
14) Zhu HJ, Patrick KS, Yuan HJ, et al. Two CES1 gene mutations lead to dysfunctional carboxylesterase 1 activity in man: clinical significance and molecular basis. Am J Hum Genet 2008:82:1241-1248
15) Zhu HJ, Appel DI, Peterson YK, et al. Identification of selected therapeutic agents as inhibitors of carboxylesterase 1: potential sources of metabolic drug interactions.
16) Berger S, Iyengar R. Network analyses in systems pharmacology. Bioinformatics 2009: 25:2466-2472.
17) Redinbo MR, Bencharit S, Potter PM. Human carboxylesterase 1: from drug metabolism to drug discovery. Biochem Soc Trans. 2003:31:620-624.
18) Crow JA, Herring KL, Xie S, et al. Inhibition of carboxylesterase activity of THP1 monocytes/macrophages and recombinant human carboxylesterase 1 by oxysterols and fatty acids. Biochim Biophys Acta 2010:1801:31-41.
19) Kaddurah-Daouk R, Krishnan KR. Metabolomics: a global biochemical approach to the study of central nervous system diseases. Neuropsychopharmacology 2009:34:173-186.
