# Supplementary material for: Outcomes of a 12-week ecologically valid observational study of first treatment with methylphenidate in a representative clinical sample of drug naïve children with ADHD
Source: PLoS One. 2021 Oct 21;16(10):e0253727. doi: 10.1371/journal.pone.0253727 (PMC8530346; doi:10.1371/journal.pone.0253727)
Supplement: S2 File — (PDF) [file pone.0253727.s012.pdf]

The relationship between CES1 genotype and methylphenidate response in children with ADHD – INDICES work package 6
Protocol version 3

Tine Houmann[1], Pia Jeppesen[1], Kerstin von Plessen[2], Anne-Katrine Pagsberg[2], Kristine Kaalund-Brok[1,] Jan Buitelaar[3] Henrik Berg Rasmussen[4]
[1]Child and Adolescent Psychiatric Center Glostrup, University of Copenhagen, Denmark, [2]Child and Adolescent Psychiatric Center Bispebjerg , University of Copenhagen, Denmark, [3]Radboud University Nijmegen Medical Center, [4]Research Institute of Biological Psychiatry, Mental Health Centre Sct. Hans, Copenhagen University Hospitals, Denmark

INDICES: (Individualised drug therapy based on pharmacogenomics: Focus on carboxylesterase1)

SUMMARY.
We wish to examine the relationship of Carboxylesterase 1(CES1) genotype with the response to Methylphenidate (MPH) in children with attention deficit hyperactivity disorder (ADHD). This will allow us to develop guidelines for individualized CES1 genotype-based MPH treatment in patients with ADHD.

The INDICES project combines pharmacology, genetics and metabolomics with clinical psychiatry and cardiology. It consists of seven work packages with focus upon carboxylesterase 1 (CES1), an enzyme with a major role in the metabolism of a variety of essential drugs including methylphenidate (MPH) and trandolapril (TA) for treatment of ADHD and cardiovascular disease (CVD), respectively.
The overall aim is to develop and implement guidelines for individualized treatments with MPH and TA and obtain a better drug response and reduce the risk of adverse reactions.

Background (Work package 6):
MPH is first-line treatment for ADHD, which affects 3-5% of children and adolescents. In most cases it persists into adulthood[1]. In Denmark the consumption of MPH has increased by a factor of 10 during the last 10 years[2]
Comorbidity is common, affecting more than 80% of patients with ADHD.
15- 20 % of patients treated with MPH are nonresponders. Adverse reactions are common, but few studies have had adverse reactions as their primary focus[3,4]. Decreased appetite is closely linked to stimulant treatment, and sleep problems occurs in approximately 70% in the initial stages of treatment. Increased blood pressure and heart rate, associated with stimulant treatment, affects a minority of treated individuals (5%). Potentially more serious cardiovascular events, however, seem to appear in stimulant treated children, with a risk, similar to the background population[5]. Discontinuation of treatment because of adverse reactions occurs in 2-10%[4]. Overall, adverse reactions in MPH treatment seem to be linked to individual underlying mechanisms, possibly related to pharmacokinetics and pharmacogenomics. Guidelines for treatment with MPH emphasize the need for individually tailored medication dosage, and further research into pharmacogenetics , but the clinical use of these tools is still highly variable[3,6,7] Several studies have found evidence for genetic variations in MPH response, especially variations in the dopamine transporter and dopamine D4 receptor. Variations in candidate genes, predict differences in ADHD treatment response and side effects of immediate release MPH[8-10]

Carboxylesterase 1(CES1) is a key enzyme in the metabolism of MPH, various other drugs, and endogenous lipids. Clinical and preclinical studies indicate that polymorphisms in the CES1 gene are responsible for individual variations in MPH treatment response and drug-drug interactions [11-13]. Recent data from a collaborative study supported by the European Commission (EU-FP7) have revealed duplication of the CES1 gene at frequencies of approximately 0.20 in Europe (Henrik Berg Rasmussen, unpublished). Gene duplication may give rise to increased rate of metabolism of CES1 dependent drugs.

Hypotheses:
Our main hypotheses: Individuals with a gene variant of CES1 resulting in rapid metabolic breakdown of MPH will experience less therapeutic effect and thus need higher dosages.
Individuals with a gene variant of CES 1 resulting in slow metabolizing of MPH will experience a better therapeutic effect and thus need lower dosages.
Our exploratory hypotheses: Dose-related side effects in MPH treatment will be associated with the metabolic rate of MPH breakdown, in line with its therapeutic effect.

Aims:
We aim to examine the influence of the CES1 genotype in children with ADHD on the effectiveness and the pharmacokinetics of MPH. Moreover, we aim to develop guidelines for individualised MPH therapy, based on CES1genotype by combining the results from INDICES Workpackage 2, a pharmacokinetic study of the correlation of CES1 genotype and copy number, with the pharmacokinetics of trandolapril and methylphenidate.
Implementation of guidelines for individualized pharmacotherapy will improve treatment efficacy and safety in this group of children.

Studypopulation:
200 drugnaïve children diagnosed with ADHD according to DSM-IV criteria in the age 7-12 years will be recruited from the three Child- and Adolescent Psychiatric Centers in the Capital Region of Copenhagen. To allow for a naturalistic setting, and because more than 80% of children and adolescents with ADHD have a comorbid psychiatric condition, we will only exclude patients with an IQ < 70, current serious medical or psychiatric illness (e.g. schizophrenia, bipolar disorder, untreated epilepsy, heart failure).

Inclusion criteria:
Age: 7-12 years (both inclusive)
Both sexes
DSM-IVADHD diagnosis (any subtype)
Clinical indication for methylphenidate treatment, based on national guidelines, confirmed by an ADHD-RS score +1.5 SD above mean adjusted for age and sex
Drug naïve to ADHD medication (Methylphenidate, Dexamphetamine, Atomoxetine)
Written informed consent by parents holding custody, and the child, to participate in the study

Exclusion criteria:
Medical conditions that contraindicate treatment with MPH: Cardiovascular or cerebrovascular disease, hyperthyroidism, phaeocromocytoma, glaucoma.
Psychiatric conditions that contraindicate treatment with MPH: Mania, psychosis, former severe depression, bipolar disorder not well controlled, suicidal conduct, anorexia nervosa

Diagnosis of mental retardation (IQ < 70)
Any ADHD medication ever (Methylphenidate, Dexamphetamine, Atomoxetine)
Current treatment with another CES1 related drug (Tamiflu, Trandolapril)
Current treatment with antipsychotics
Treatment with irreversible MAO inhibitors within the last 14 days before treatment start

Methods:
The psychometric instruments used in this study, for diagnostic assessment, are standard procedures for children in the age group, referred with a possible diagnosis of ADHD, in the Capital Region of Copenhagen. The staffs (Child- and adolescent psychiatrists and psychologists) who are conducting the assessments at the three study sites have all been trained in the psychometric instruments used in this study. We will measure interrater-reliability (by using kappa) for the K-SADS interviews.
The INDICES work package 6 study group includes a local child and adolescent psychiatrist, who is the responsible researcher on each of the three study sites.

Diagnostic assessment:
All children referred to the three Centers of Child and Adolescent Psychiatry in the Capital Region of Copenhagen, with a possible diagnosis of ADHD, are assessed by the following procedures:
The CBCL[14] (web-based questionnaires)
K-SADS interview[15]
Clinical assessment and diagnosis of ADHD according to Danish national guidelines, including history of development, symptoms and sociodemographics, clinical observation of symptoms, cognitive test and standard physical examination
ADHD diagnosis confirmed by K-SADS
Comorbidity diagnosed with K-SADS.
Possible cases, based on age and ADHD diagnosis, are evaluated by the local responsible researcher for inclusion in the study, and if relevant, contacted for permission and written informed consent by parent(s) holding custody, and the child, to participate in the study

Baseline ratings and measures on patients meeting inclusion criteria:
ADHD-RS [16] rated by parent
ADHD-RS rated by teacher
Investigator rated ADHD-DSM-IV-RS [17]
CGI- Severity scale [18]
Connors CPT[19] or TOVA[20](to be finally decided)
Adverse reactions-Rating Scale [21]
ASK-ME parent rated questionnaire, attitude scale (7 questions) [22]
Body weight and height
Blood pressure and pulse

DNA samples:
A sample of saliva

Patients included in the study enter a twelve-week study period with dose escalation of methylphenidate, weekly telephone ratings and monthly clinical assessments of effect and adverse reactions to MPH treatment. Weekly telephone ratings are done by the PhD. student, and the following decision on pursuing further dose escalation is made in cooperation with the

child's regular psychiatrist at the study site. Monthly clinical assessments and decisions on dose escalation are done by the child's regular psychiatrist.

Weekly assessment and ratings:
(not made in week 4, 8 and 12)
By telephone: ADHD-DSM-IV-RS and Adverse Reaction-Rating Scale .
Weekly decision on pursuing further dose escalation based on the above ratings

Clinical assessment:
Every four weeks:
Measurement of blood pressure, pulse, weight and height
ADHD-DSM-IV-RS
Parent rated ADHD-RS
Adverse reactions-Rating Scale

Supplementary rating and test at week 8:
Teacher rated ADHD-RS
Blood test to measure serum concentration of Ritalin acid, and DNA

Outcome ratings after twelve weeks :
Investigator rated ADHD-DSM-IV-RS (primary outcome measure)
CGI-Severity (secondary outcome measure)
CGI-Improvement (secondary outcome measure)
CGI-Efficacy (secondary outcome measure)
Connors CPT or TOVA(secondary outcome measure)
Parent rated ADHD-RS (secondary outcome measure)
Teacher rated ADHD-RS (secondary outcome measure)
Adverse reactions-Rating Scale (secondary outcome measure)

Outcome measures:
Investigator rated ADHD-DSM-IV-RS score
Parent rated ADHD-RS score
Teacher rated ADHD-RS score
CGI-I
CGI-S
CGI-E
Connors or TOVA
MPH dose required for "Borderline normalisation" on ADHD-DSM-IV-RS: T-score 60-70
MPH dose required for normalisation on ADHD-DSM-IV-RS: T-score < 60
Occurrence (no. of weeks from treatment initiation) and type of adverse reactions
MPH dose at occurrence of adverse reaction
MPH dose at discontinuation, if that is necessary, because of adverse drug reactions or Treatment failure (no effect of MPH on ADHD core symptoms)
Serum concentration of Ritalin acid at week 8

Body weight, height, blood pressure and pulse

Dose escalation:
MPH is initiated at a low dose: 5mg. MPH-IR three times daily (TID) and escalated weekly with 0 - 2,5 - 5mg MPH-IR TID. Maximum dose is 2mg/kg/day. MPH dose morning, midday and afternoon can be individually escalated, based on effect and adverse reactions. Decisions on dose escalation will be based on a clinical decision manual, elaborated for the study. Individual deviations in dose escalation are noted in the CRF and case records.

Dose escalation is continued on a weekly basis until one of the following:

- Normalisation on ADHD-RS defined as T-score < 60
- Borderline normalisation on ADHD-RS, if normalisation is not obtainable, defined as T-score 60-70
- Cessation of dose escalation because of adverse reactions
- Discontinuation of MPH because of either intolerable adverse reactions, or no effect of MPH on ADHD core symptoms.

On basis of this, we exploratively will categorize patients as

1) Clinically normal reponders
2) Clinically slow responders
3) Clinically rapid responders

Results:
We plan to use multivariate regression models with the change in symptom severity, measured with the ADHD-DSM-IV rating scale score as the dependent measure, whereas CES1genotype, weight adjusted MPH dosage (mg/kg/day), age, gender, occurrence of adverse reactions and ADHD subtype will serve as predictors.
We will repeat the analysis with CGI-I as the dependent measure.
This will allow us to assess whether MPH is less effective in subjects with CES1 gene variants, that are known to have a rapid metabolic breakdown of MPH, whether higher dosages of MPH, necessary to adapt to the genetic differences in these subjects, are associated with more side effects, whether subjects with CES1 gene variants that are known to have a slow metabolic breakdown of MPH, need lower dosages of MPH than expected, to have effect on ADHD core symptoms, and whether lower dosages of MPH, necessary to adapt to the genetic differences in these subjects, are associated with less side effects.
By combining our results with data from the pharmacokinetic work-package in the INDICES study, we will develop guidelines for individualised therapy of ADHD with MPH

Ethics
The clinical work package 6 focused on ADHD is covered by existing permissions from the local committees on scientific ethics (De Videnskabsetiske Komiteer for Region Hovedstaden) and the Danish Data Protection Agency (Datatilsynet). Those permissions allow the collection and examination of data as well as biological samples from psychiatric patients with ages below 18 years, for research purposes, including genetic research projects. Journal number: H-B-2009-026

Participating centres and collaborators:
Child and Adolescent psychiatric Center Glostrup, Copenhagen University Hospitals:
Tine Houmann (TH), Senior Consultant, Associate Professor, Pia Jeppesen (PJ), PhD, Post Doc, Senior consultant, Associate Professor, Kristine Kaalund-Brok (KKB) MD
Child and Adolescent psychiatric Center Bispebjerg, Copenhagen University Hospitals:
Kerstin von Plessen (KP), PhD, Senior Consultant, Professor, Katrine Pagsberg (KP), PhD, Senior consultant, Associate Professor
Child and Adolescent psychiatric Center Hillerød, Copenhagen University Hospitals:
Jørgen Dyrborg (JD) Senior Consultant, Maj-Britt Lauritsen (MBL) MD
Radboud University Nijmegen Medical Center:
Jan K. Buitelaar (JB) Professor
Research Institute of Biological Psychiatry, Psychiatry Center Sct. Hans, Copenhagen University Hospitals:
Henrik Berg Rasmussen (HBR) PhD, Post doc

Milestones
2012: Month 1-2: planning and introduction
2012: Month 3-12: recruitment of 100 patients who will complete a 3-month study period of drugescalation
2013: Month 13-24: recruitment of 100 additional patients who will complete a 3-month study period corresponding to a total of 200
2014: Month 25-29: Finalization of entire clinical study. Data analysis. Preparation and submission of two manuscripts.
2014: Month 30-36: study visit at University of Nijmegen
2015: Month 37-39: Statistical treatment of data and elaboration of manuscripts. Advices for improved stimulant therapy developed
2015: Month 40-42: Preparation of PhD thesis. (Month 29-42: preparation of PhD thesis)

Flowchart medication trial:

Assessments by telephone, Week 1,2,3,5,6,7,9,10,11:
ADHD-DSM-IV-RS
Adverse reaction-RS

Clinical assessment week 4:
Weight, height, pulse, blood pressure
ADHD-DSM-IV-RS
Parent rated ADHD-RS
Adverse reactions-RS

Clinical assessment week 8+12:
Weight, height, pulse, blood pressure
ADHD-DSM-IV-RS
Parent rated ADHD-RS
Teacher rated ADHD-RS
Adverse reactions-RS
Only week 8:Blood test (Se.Ritalin

MPH-IR 5mg x 3

Dose escalation:
MPH- IR (0-2.5-5 mg x3)
weekly

Discontinuation of MPH because of intolerable adverse reactions

Discontinuation of dose escalation because of adverse reactions

Discontinuation of MPH
Because of treatment faillure

ADHD-RS borderline symptom – normalisation T-score 60-70

Discontinuation of dose escalation because of adverse reactions

ADHD-RS symptom normalisation
T-score$\leq$ 60 (norm meanscore +1SD)

Outcome ratings:
Investigator rated ADHD-DSM-IV-RS, CGI-I, CGI-S, parent rated ADHD-RS, teacher rated ADHD-RS, Connors-CPT/TOVA, Adverse reactions –RS

Reference List

(1) Pliszka S, AACAP work group.
Practice parameter for the assessment and treatment of children and adolescents with attention-deficit/hyperkinetic disorder.
2007
J Am Acad Child Adolesc Psychiatry 2007;4:894-921.

(2) Danish Medicine Agency. MedStat. www laegemiddelstyrelsen dk, 2011Available from: URL: www.laegemiddelstyrelsen.dk,

(3) Graham J, Banaschewski T, Buitelaar J, Coghill D, Danckaerts M, Dittmann RW, et al.
European guidelines on managing adverse effects of medication for ADHD.
2011
Eur Child Adolesc Psychiatry 2011;20(1):17-37.

(4) Graham J, Coghill D.
Adverse effects of pharmacotherapies for attention-deficit hyperactivity disorder: epidemiology, prevention and management.
2008
CNS Drugs 2008;22(3):213-37.

(5) Hammerness PG, Perrin JM, Shelley-Abrahamson R, Wilens TE.
Cardiovascular risk of stimulant treatment in pediatric attention-deficit/hyperactivity disorder: update and clinical recommendations.
2011
J Am Acad Child Adolesc Psychiatry 2011;50(10):978-90.

(6) Pliszka S, AACAP work group.
Practice parameter for the assessment and treatment of children and adolescents with attention-deficit/hyperkinetic disorder.
2007
J Am Acad Child Adolesc Psychiatry 2007;4:894-921.

(7) Powell SG, Thomsen PH, Frydenberg M, Rasmussen H.
Long-term treatment of ADHD with stimulants: a large observational study of real-life patients.
2011
J Atten Disord 2011;15(6):439-51.

(8) Froehlich TE, McGough JJ, Stein MA.
Progress and promise of attention-deficit hyperactivity disorder pharmacogenetics.
2010
CNS Drugs 2010;24(2):99-117.

(9) Froehlich TE, Epstein JN, Nick TG, Melguizo Castro MS, Stein MA, Brinkman WB, et al.
Pharmacogenetic predictors of methylphenidate dose-response in attention-

deficit/hyperactivity disorder.
2011
J Am Acad Child Adolesc Psychiatry 2011;50(11):1129-39.

(10) McGough JJ, McCracken JT, Loo SK, Manganiello M, Leung MC, Tietjens JR, et al.
A candidate gene analysis of methylphenidate response in attention-deficit/hyperactivity disorder.
2009
J Am Acad Child Adolesc Psychiatry 2009;48(12):1155-64.

(11) Nemoda Z, Angyal N, Tarnok Z, Gadoros J, Sasvari-Szekely M.
Carboxylesterase 1 gene polymorphism and methylphenidate response in ADHD.
2009
Neuropharmacology 2009;57(7-8):731-3.

(12) Zhu HJ, Appel DI, Peterson YK, Wang Z, Markowitz JS.
Identification of selected therapeutic agents as inhibitors of carboxylesterase 1: potential sources of metabolic drug interactions.
2010
Toxicology 2010;270(2-3):59-65.

(13) Xie S, Borazjani A, Hatfield MJ, Edwards CC, Potter PM, Ross MK.
Inactivation of Lipid Glyceryl Ester Metabolism in Human THP1 Monocytes/Macrophages by Activated Organophosphorus Insecticides: Role of Carboxylesterases 1 and 2.
2010
Chem Res Toxicol 2010.

(14) Achenbach T.M. Manual for the child Behavior Checklist/4-18 and 1991 Profile. Burlington VT: University of Vermont Department of Psychiatry; 1991.

(15) Ambrosini PJ.
Historical development and present status of the schedule for affective disorders and schizophrenia for school-age children (K-SADS).
2000
J Am Acad Child Adolesc Psychiatry 2000;39(1):49-58.

(16) DuPaul G, Power T, McGoey K, Ikeda M, Anastopoulos A. AD/HD Rating Scale IV: Checklists, norms, and clinical interpretation. New York: Guilford; 1998.

(17) Zhang S, Faries DE, Vowles M, Michelson D.
ADHD Rating Scale IV: psychometric properties from a multinational study as a clinician-administered instrument.
2005
Int J Methods Psychiatr Res 2005;14(4):186-201.

(18) Berk M, Ng F, Dodd S, Callaly T, Campbell S, Bernardo M, et al.
The validity of the CGI severity and improvement scales as measures of clinical effectiveness suitable for routine clinical use.

2008
J Eval Clin Pract 2008;14(6):979-83.

(19) Conners CK, Epstein JN, Angold A, Klaric J.
Continuous performance test performance in a normative epidemiological sample.
2003
J Abnorm Child Psychol 2003;31(5):555-62.

(20) Schatz AM, Ballantyne AO, Trauner DA.
Sensitivity and specificity of a computerized test of attention in the diagnosis of Attention-Deficit/Hyperactivity Disorder.
2001
Assessment 2001;8(4):357-65.

(21) Barkley RA, McMurray MB, Edelbrock CS, Robbins K.
Side effects of methylphenidate in children with attention deficit hyperactivity disorder: a systemic, placebo-controlled evaluation.
1990
Pediatrics 1990;86(2):184-92.

(22) Dosreis S, Zito JM, Safer DJ, Soeken KL, Mitchell JW, Jr., Ellwood LC.
Parental perceptions and satisfaction with stimulant medication for attention-deficit hyperactivity disorder.
2003
J Dev Behav Pediatr 2003;24(3):155-62.
