# Supplementary material for: Outcomes of a 12-week ecologically valid observational study of first treatment with methylphenidate in a representative clinical sample of drug naïve children with ADHD
Source: PLoS One. 2021 Oct 21;16(10):e0253727. doi: 10.1371/journal.pone.0253727 (PMC8530346; doi:10.1371/journal.pone.0253727)
Supplement: S3 Table — (PDF) [file pone.0253727.s004.pdf]

S3 Table. Comparison of ADHD core symptoms severity between included and excluded patients

ADHD symptoms before inclusion	Included (n = 207) M (SD)	Excluded (n = 211) M (SD)	Included versus excluded			Refused medicine (n = 95) M (SD)	Included versus refused medicine		
			**M dif. (SD dif.)**	**95% CI**	***p*-value**		**M dif. (SD dif.)**	**95% CI**	***p*-value**
**Parent**									
**Inattention**	17.4[1] (5.05)	15.1[7] (5.6)	2.3 (0.6)	(1.3, 3.4)	< 0.001	14.6[12] (5.3)	2.8 (0.7)	(1.5, 4.1)	< 0.001
**Hyperactivity-Impulsivity**	15.3[2] (6.1)	12.5[7] (6.0)	2.9 (0.6)	(1.6, 4.1)	< 0.001	10.9[13] (5.3)	4.5 (0.8)	(3.0, 6.0)	< 0.001
**Conduct problems**	10.5[3] (5.9)	8.1[8] (5.5)	2.4 (0.6)	(1.3, 3.6)	< 0.001	7.5[14] (5.1)	3.0 (0.7)	(1.6, 4.5)	< 0.001
**Teacher**									
**Inattention**	17.1[4] (4.9)	18.5[9] (5.1)	-1.4 (0.5)	(-2.4, -0.3)	0.010	18.2[15] (4.6)	-1.1 (0.6)	(-2.4, 0.1)	0.081
**Hyperactivity-Impulsivity**	9.0[5] (5.7)	15.1[10] (7.2)	-6.0 (0.7)	(-7.4, -4.7)	< 0.001	14.3[16] (7.3)	-5.2 (0.8)	(3.6, 6.8)	< 0.001
**Conduct problems**	4.5[6] (4.6)	9.0[11] (6.5)	-4.4 (0.6)	(-5.6, -3.3)	< 0.001	8.2[17] (6.4)	-3.6 (0.7)	(-5.0, 2.3)	< 0.001

Independent t-test. Comparison between included vs. excluded patients and comparison between included and patients excluded due to parent's decision of refusing medicine (a subgroup of excluded patients)

M = Mean. M dif. = Mean difference. SD dif. = Standard deviation difference. *n* = number.

ADHD-Rating Scale (DuPaul). Inattention subscale: 9 items [range 0-27]. Hyperactivity-Impulsivity subscale: 9 items [range 0-27]. Conduct problems subscale: 8 items [range 0-24]. Rated by parent (ADHD-RS-P) or teacher (ADHD-RS-T).

Number of participants with observed outcome data: [1] *n* = 200, [2] *n* = 197, [3] *n* = 204, [4] *n* = 187, [5] *n* = 191, [6] *n* = 193, [7] *n* = 174, [8] *n* = 181, [9] *n* = 170, [10] *n* = 173, [11] *n* = 177, [12] *n* = 78, [13] *n* = 80, [14] *n* = 84, [15] *n* = 78, [16] *n* = 77, [17] *n* = 79.
