# Supplementary material for: Outcomes of a 12-week ecologically valid observational study of first treatment with methylphenidate in a representative clinical sample of drug naïve children with ADHD
Source: PLoS One. 2021 Oct 21;16(10):e0253727. doi: 10.1371/journal.pone.0253727 (PMC8530346; doi:10.1371/journal.pone.0253727)
Supplement: Attachment — Submitted filename: Response to reviewers_17_01_21.docx [file pone.0253727.s013.docx]

Dear Andrea Martinuzzi, Academic Editor, PLOS ONE

Thank you for the opportunity to resubmit our manuscript for further consideration in PLOS ONE. We are very thankful for the peer reviewer’s effort and time taken to review our manuscript. We very much appreciate the comments and the suggestions that have helped us clarify certain points and further improve the quality of our manuscript. Please find below our response to the reviewer comments.

We hope that the revised version is now acceptable for publication in PLOS One and look forward to hearing from you.

Sincerely,

Kristine Kaalund-Brok,

on behalf of the group of authors

In your revised cover letter, please address the following prompts:

1. Please ensure that your manuscript meets PLOS ONE's style requirements, including those for file naming. The manuscript meets PLOS ONE's style requirements now.

2. We include Data Availability statement.

3. One of the noted authors is a group or consortium [INDICES]. A lead author for this group along with a contact email address is now provided on the top of “List of all partners in the INDICES Consortium”.

4. We note that you have included the phrase “data not shown” in your manuscript. All data are available in the text now.

5. Please include a copy of Table 10 which you refer to in your text on page 20. This was misleading, and it is now corrected to Table 4.

1. Is the manuscript technically sound, and do the data support the conclusions?

The manuscript must describe a technically sound piece of scientific research with data that supports the conclusions. Experiments must have been conducted rigorously, with appropriate controls, replication, and sample sizes. The conclusions must be drawn appropriately based on the data presented.

Reviewer #1: Partly

Reviewer #2: Yes

Reviewer #3: Partly

2. Has the statistical analysis been performed appropriately and rigorously?

Reviewer #1: No

Reviewer #2: Yes

Reviewer #3: Yes

3. Have the authors made all data underlying the findings in their manuscript fully available?

The [PLOS Data policy](http://www.plosone.org/static/policies.action#sharing) requires authors to make all data underlying the findings described in their manuscript fully available without restriction, with rare exception (please refer to the Data Availability Statement in the manuscript PDF file). The data should be provided as part of the manuscript or its supporting information, or deposited to a public repository. For example, in addition to summary statistics, the data points behind means, medians and variance measures should be available. If there are restrictions on publicly sharing data—e.g. participant privacy or use of data from a third party—those must be specified.

Reviewer #1: Yes

Reviewer #2: Yes

Reviewer #3: Yes

4. Is the manuscript presented in an intelligible fashion and written in standard English?

PLOS ONE does not copyedit accepted manuscripts, so the language in submitted articles must be clear, correct, and unambiguous. Any typographical or grammatical errors should be corrected at revision, so please note any specific errors here.

Reviewer #1: Yes

Reviewer #2: Yes

Reviewer #3: Yes

5. Review Comments

Review Comments to the Author

Please use the space provided to explain your answers to the questions above. You may also include additional comments for the author, including concerns about dual publication, research ethics, or publication ethics. (Please upload your review as an attachment if it exceeds 20,000 characters)

Reviewer #1: The manuscript entitled ‘Outcomes of a 12-week ecologically valid observational study of first treatment with methylphenidate in a representative clinical sample of drug naïve children with ADHD’ with the aim to describe treatment responses and their predictors during the first 12 weeks of MPH treatment using repeated measurements of symptoms and adverse reactions (ARs) to treatment in 207 children recently diagnosed with ADHD.

The manuscript can be further improved based on the following comments.


Study Design
Page 6 the sentence ‘There was made a protocol (S11 and S12)’ to be revised.*Reply: This is revised. It now reads: Study protocol is available (S11 and S12*)

Page 7, the statement ‘the study was not registered in a registry for clinical trials (see the ethics section)’ not clear. In the ethics section, it was written ‘The study was registered in ClinicalTrials.gov (NCT04366609)’.

*Reply: This is now revised. The misleading sentence is now deleted.*

CGI-S, CGI-I, WFIRS-P, BSSERS-C language version and scoring method including classification of scores to be highlighted.

*Reply: The language versions are now added. Which rater who scored was defined. It now reads:*

- *The clinician-rated CGI-S (English version) is a seven-point Likert scale, rated 1 (normal, not ill at all) to 7 (among the most extremely ill patients), used to measure the global severity of symptoms and functional impairments of mental health disorder including ADHD and psychiatric comorbidities. Consensus ratings were performed in week 0 and 12.* *A beneficial symptom reduction was defined by a CGI-S score of 1 or 2 (normal, not ill at all or borderline ill) based on a definition from another study [30]*
- *The clinician-rated CGI-I (English version) is also a seven-point Likert scale rated from 1 (very much improved) to 7 (very much worse). Consensus ratings were performed in week 12.* *A beneficial symptom reduction was defined by a CGI-I score of 1 or 2 (very much improved or much improved) based on a definition from other studies [33,39,40].*
- *WFIRS-P (Danish version) is a parent-rated questionnaire, with 50 questions about a child’s daily and social functioning in six different domains of a child’s life: family (10 items), learning and school (10 items), activities of daily living (10 items), self-concept (3 items), social activities (7 items), and risky activities (10 items). It is evaluated on a four-point Likert scale from 0 (never or not at all) to 3 (very often or very much). Index scores of WFIRS-P were measured in week 0 and 12. Single item scores = 2-3 signal impairment (> 2 SD).* *If the parent-reported scores were missing for a single item on a subscale (maximum 1 item) we still calculated the index score as the sum-score divided by the number of items that were scored.*
- *ARs were measured by the clinical investigator using the BSSERS-C (Danish version).*

*The BSSERS-C scoring system was weakly described in the literature. This study elaborated its own definition and graduation for each adverse reaction. The study did not define a sever adverse reaction – but post hoc a definition of server reduced appetite was made.*

Page 11, for the statement ‘Ten percent missing data on items on WFIRS-P subscales were allowed and missing data on items were set as 0 (never or not at all)’ reason to be provided. Description on the missing data to be provided. i.e percentages of missing data, pattern etc.

*Reply: WFIRS-P was parent-rated therefore there were some missing data, if the parents did not answer all questions and at the same time the clinician was not aware of the missing data therefore did not ask the parents about the missing data. See S7 Table for missing data.*

Page 11 symbol X² to be replaced with symbol 'Chi' square. Likewise with S9 Table footnote (italicize X to be replaced with 'Chi' symbol).

*Reply: This is now revised.*

Page 12, the citation for SPSS including publisher name to be highlighted.

*Reply: This is now revised.*

Results
Page 12 for ‘187 (90.3%) patients (mean age 9.6 (SD 1.5)’ the mean and SD are similar to mean, sd for 207 patients.

*Reply: Yes, the mean ages and SDs are the same.*

Figure 1, difficult to be visualized.

*Reply: This Figure 1 is now easier to visualize.*

Page 13 Table 1, decimal points to be standardized. 9.88 (comma to be replaced with dot)

*Reply: This is now revised*.

Page 16 Table 2, bp/m to be written as bpm. Effect size could be presented.

*Reply: bpm is now revised and effect sizes are now presented.*

Page 15 for the statement ‘The mean percent reductions of scores on the clinician-rated ADHD-RS-C subscale from week 0 to 12 were 52.0% on Inattention and 56.0% on Hyperactivity-Impulsivity' 52.0 % and 56.0% to be revised as 51.7% and 53.0%.

*Reply: This is now revised.*

Page 15, for the statement ‘ nd the mean percentage reductions on the parent-rated ADHD-RS-P subscales were 48.1% on Inattention, 45.0% on Hyperactivity-Impulsivity, and 50.7% on Conduct problems (S6 Table)’ the figures did not tally with the figures in S6Table.

*Reply: This is now revised.*

Page 15, for ‘CI 95% 2.2 to 2.5’ & Page 17 ‘CI 95% 0.5 to 0.9), Page 19 & 21 text and Table 4 & 5' CI95% to be written as 95%CI.

*Reply: This is now revised to 95%CI everywhere.*

Page 17, Adverse reactions, Table 2 to be cited for height and weight findings.

*Reply: It is citated at the page in the last sentence in the part of adverse reactions.*

Page 18 Table 3, what estimate refers, to be clearly labelled/defined.

*Reply: This is now revised.*

Page 18 Table 3, Page 20 Table 4, Page 21 Table 5, model fit/goodness of fit measures to be stated.

*Reply: All outcomes where normal distributed in week 0 and in week 12 and model fit indicated acceptable fit of the selected normal distributed models with apart from sum score of reduced appetite which where dichotomized.*

Page 21 Table 5, the estimate to be replaced with b. SD errors to be written as std error.

*Reply: This is now revised.*

Page 21, for the statement 'Severely ill and most extremely ill rated patients (CGI-S 6-7) had a significantly higher risk of reduced appetite compared with patients rated not ill to moderately ill (CGI-S 1-5) in week 0 and throughout the whole treatment period (OR = 2.41, CI 95% 1.42 to 4.08).' the results to be displayed in the table.

*Reply: This is now displayed in the Table 3*

Page 21, for the statement 'Higher CGI-S scores at week 0 predicted a higher end dose of IR-MPH. CGI-S at week 0 and age explained together 12.7% (p > 0.001) of the variance of the end-dose IR-MPH' indicate clearly R square and denoted in the table footnote. For p >0.001, to use < and representative p value.

*Reply: This is now displayed in the Table 5 and is revised in the text.*

S4 Table footnote, were all these confirmed missing data and not numbers after minus the missing data? Missing data: ¹ n = 147, ² n= 143, ³ n = 195, ⁴ n = 193; S5 Table footnote Missing data: ¹ n = 66, ² n = 48, ³ n = 59; S6 Table footnote Missing data: ¹ n = 174, ² n = 171, ³ n = 163, ⁴ n = 179, ⁵ n = 127, ⁶. Likewise please check S7 Table and S8 Table.

*Reply: Thank you for pointing out this mistake, we meant the opposite: It now reads: Number of participants with observed outcome data.*

S4 Table, for 'Medium, higher education (15-14 years)' should be written as 15-16 years.

*Reply: This is now revised.*

S4 Table, Cap for 'T'otal. Likewise for total in S7 Table.

*Reply: This is now revised.*

S9 Table 9, symbol % to be highlighted in the table.

*Reply: This is now revised.*

S5 Table, symbol % for individual figure to be omitted since the symbol was highlighted after the variable name. Likewise with S6 Table.

*Reply: This is now revised.*

Limitation on sample size or power of study to be discussed if any.

*Reply:* The sample size was adequate for the objectives of the study, as noted by reveiwer 3. We added the following text to the article, page 11: *The study was powered to detect effects of genetic variants on outcome regarding the end-dose of IR-MPH = 1 mg/kg/day (SD 0.5 mg/kg/day) and the average MPH titration duration = 6 weeks (SD 2 weeks). Setting a significance level of 0.05 (one-sided for genetic variants) and a power of 0.80, then a minimum relevant effect measured as a regression-coefficient of β = 0.25 (mg/kg/day) or β = 0.1 (weeks) would be detectable in 94 children with a frequency of 0.20 of the genetic variant of interest. It was decided to include at least 200 children to be able to study also less frequent variants with a relatively large impact on the outcome.*

Reviewer #2: This is a timely and well conducted study. The rationale is presented in a very clear way and the authors make good points in the need for more ecologic valid studies as well as the lack of a standard definition of "response" in the field. the methods are appropriate and the reporting good. I only have a few suggestions:
“Attention deficit/hyperactivity disorder (ADHD) is a heterogeneous neurodevelopmental disorder characterised by pervasive and impairing symptoms of inattention and/or hyperactivity and impulsivity with onset of symptoms before age 7 years (International Classification of Diseases and Related Health Problems, ICD-10)[1]” is per se not correct as ICD -10 does not include ADHGD; rather, it includes Hyperkinetic syndrome, which is roughly equivalent to the ADHD combined presentation of the DSM-5. So the authors may rephrase as follows (or similar): “Attention deficit/hyperactivity disorder (ADHD) as defined in the DSMS is a heterogeneous neurodevelopmental disorder characterised by pervasive and impairing symptoms of inattention and/or hyperactivity and impulsivity with onset of symptoms before age 12 or 7 when considering the definition of Hyperkinetic syndrome (International Classification of Diseases and Related Health Problems, ICD-10)[1], roughly equivalent to the combined presentation of ADHD as per the DSM”.

*Reply: Thank you for this helpful comment and suggestion. We agree. It now reads:*

*Attention deficit/hyperactivity disorder (ADHD) as defined in the DSM-IV/-5 (Diagnostic and Statistical Manual of Mental Disorders, DSM)[1] is a heterogeneous neurodevelopmental disorder characterised by pervasive and impairing symptoms of inattention and/or hyperactivity and impulsivity with onset of symptoms before age 7 (DSM-IV) or age 12 (DSM-5). The syndrome of Hyperkinetic disorder based on the International Classification of Diseases and Related Health Problems (ICD-10)[2] is roughly equivalent to the combined presentation of ADHD as per the DSM-IV/-5, and* *regardless of definitions, the ADHD syndromes show high rates of persistence into adulthood[3,4]. ……*

The focus of the paper ins on the pharmacological treatment … but the author may want to remind the reader that non pharmacological treatments, in particular parent training behaviors, offer complementary interventions (eg, for opposition behaviors) (eg: <https://pubmed.ncbi.nlm.nih.gov/29083042/> )

*Reply: Thank you very much for this suggestion.*

*We added: ..and recent guideline strongly recommend parent management training and other behavioural treatments within family and school along with medication[8,9].*

*We added the following references:*

- *Wolraich ML, Hagan JF, Allan C, Chan E, Davison D, Earls M, et al. Clinical Practice Guideline for the Diagnosis, Evaluation, and Treatment of Attention-Deficit/Hyperactivity Disorder in Children and Adolescents. Pediatrics. 2019;144. doi:10.1542/peds.2019-2528*
- *Daley D, Oord SVD, Ferrin M, Cortese S, Danckaerts M, Doepfner M, et al. Practitioner Review: Current best practice in the use of parent training and other behavioural interventions in the treatment of children and adolescents with attention deficit hyperactivity disorder. J Child Psychol Psychiatry. 2018;59: 932–947. doi:https://doi.org/10.1111/jcpp.12825*

“Several meta-analyses of the short-term efficacy of immediate release MPH (IR-MPH) in randomized placebo-controlled trials have reported large effect sizes (range from 0.54 to 1.83) on ADHD core symptoms, when effects are measured as differences in endpoint or change in scores of parent-rated and teacher-rated ADHD symptoms and behaviour”: please provide references

*Reply: Several meta-analyses of the short-term efficacy of immediate release MPH (IR-MPH) in randomized placebo-controlled trials have reported large effect sizes (range from 0.54 to 1.78) on ADHD core symptoms, when effects are measured as differences in endpoint or change in scores of parent-rated and teacher-rated ADHD symptoms and behaviours.*

*The references are now provided: We added the following references:*

- *Schachter HM, Pham B, King J, Langford S, Moher D. How efficacious and safe is short-acting methylphenidate for the treatment of attention-deficit disorder in children and adolescents? A meta-analysis. CMAJ Can Med Assoc J J Assoc Medicale Can. 2001;165: 1475–1488.*
- *Storebø OJ, Ramstad E, Krogh HB, Nilausen TD, Skoog M, Holmskov M, et al. Methylphenidate for children and adolescents with attention deficit hyperactivity disorder (ADHD). Cochrane Database Syst Rev. 2015; CD009885. doi:10.1002/14651858.CD009885.pub2*

Methods
“There was made a protocol (S11 and S12)” is a bit odd; I would suggest. Study protocol is available (S11 and S12)

*Reply: This is now revised.*

Results
I wonder whether the normalisation, in addition to /borderline normalisation results could be also presented (including in the abstract)

*Reply: The differentiation between normalisation and borderline normalization is now added to the result section. It now reads:* *A total of 137 (73.2%) patients obtained Nor/Bnor (70 (37.4%) patients obtained Nor) on the Inattention subscale (ADHD-RS-C) at week 12 (Nor and Bnor are separated in S5 Table). Of these patients, 111 (81.0%) were boys and 89 (65.0%) were 7 to 9 years of age. A total of 157 (84.0%) patients obtained Nor/Bnor (95 (58,8% patients obtained Nor) on the Hyperactivity-Impulsivity subscale (ADHD-RS-C) at week 12.*

Discussion
The lack of blinding should also be discussed among the limitations.

*Reply: the discussion now includes:* *The limitations of this observational study included lack of a control condition. Furthermore, the assessors could not be blinded to dose of MPH, because the dosing was individually titrated based on the weekly assessments of child’s effects and ARs.*

Reviewer #3: The paper focuses on one of the most common psychiatric diseases in childhood and adolescence. This fact makes it particularly interesting and current. The drug therapy of ADHD and the risk / benefit relationship between therapy and adverse reactions are a permanent topic of discussion in the scientific community. Therefore this paper addresses the two most controversial points: the therapeutic efficacy of methylphenidate and its safety.

*Reply: We thank the reviewer for recognizing the importance of the topic.*

Clinicians, statisticians and epidemiologists consider time to be one of the main variables in observational and experimental studies. From this point of view, the choice to make an ecological study of only 12 weeks creates some perplexity. One of the critical points of ADHD drug therapy is maintaining efficacy over time. In the MTA study, also cited by the authors of this paper, a decrease in the therapeutic response was observed between the second and third year of treatment. The safety evaluation of a drug also takes a long time, especially for the observation of rare adverse events.
The study design and methodology used are very good and the statistical analysis is of a remarkable level. The choice of predictors of the therapeutic response is accurate. The sample size is adequate for the objectives of the study.

*Reply: We thank the reviewer for the positive comments and the raising the important discussion of the long-term outcome of MPH treatment. The current paper present data regarding the 12 weeks follow-up, while the next report from this study will focus on long-term outcome, including register-based prescription data to describe the continuation/discontinuation of MPH use over a 3-year follow-up period. Unfortunately, at the current point in time, these data have not yet been analysed and prepared for publication. We added the following comment to the research perspectives:* *Studies of effects and adverse reactions to long-term treatment with methylphenidate are warrened as these observations are lacking in the literature.*

Other comments:

*Methods, page 7*: *The clinical investigator and the children’s regular clinicians where not blind to the dosage of IR-MPH, as they collaborated to personalise the dosing of MPH based on the weekly assessments of the ADHD core symptoms and ARs for the treatment of each child.*
