# Supplementary material for: Outcomes of a 12-week ecologically valid observational study of first treatment with methylphenidate in a representative clinical sample of drug naïve children with ADHD
Source: PLoS One. 2021 Oct 21;16(10):e0253727. doi: 10.1371/journal.pone.0253727 (PMC8530346; doi:10.1371/journal.pone.0253727)
Supplement: Attachment — Submitted filename: Response to reviewer_31__05_21.docx [file pone.0253727.s014.docx]

PONE-D-20-01564R1
Outcomes of a 12-week ecologically valid observational study of first treatment with methylphenidate in a representative clinical sample of drug naïve children with ADHD.
PLOS ONE

Dear Dr. Kaalund-Brok,

Thank you for submitting your manuscript to PLOS ONE. After careful consideration, we feel that it has merit but does not fully meet PLOS ONE’s publication criteria as it currently stands. Therefore, we invite you to submit a revised version of the manuscript that addresses the points raised during the review process.

Please address the few remaining concerns of reviewer 2.

Please submit your revised manuscript by April 30th. If you will need more time than this to complete your revisions, please reply to this message or contact the journal office at [plosone@plos.org](mailto:plosone@plos.org). When you're ready to submit your revision, log on to <https://www.editorialmanager.com/pone/> and select the 'Submissions Needing Revision' folder to locate your manuscript file.

Please include the following items when submitting your revised manuscript:

- A rebuttal letter that responds to each point raised by the academic editor and reviewer(s). You should upload this letter as a separate file labeled 'Response to Reviewers'.
- A marked-up copy of your manuscript that highlights changes made to the original version. You should upload this as a separate file labeled 'Revised Manuscript with Track Changes'.
- An unmarked version of your revised paper without tracked changes. You should upload this as a separate file labeled 'Manuscript'.

If you would like to make changes to your financial disclosure, please include your updated statement in your cover letter. Guidelines for resubmitting your figure files are available below the reviewer comments at the end of this letter.

If applicable, we recommend that you deposit your laboratory protocols in protocols.io to enhance the reproducibility of your results. Protocols.io assigns your protocol its own identifier (DOI) so that it can be cited independently in the future. For instructions see: <http://journals.plos.org/plosone/s/submission-guidelines#loc-laboratory-protocols>. Additionally, PLOS ONE offers an option for publishing peer-reviewed Lab Protocol articles, which describe protocols hosted on protocols.io. Read more information on sharing protocols at <https://plos.org/protocols?utm_medium=editorial-email&utm_source=authorletters&utm_campaign=protocols>.

We look forward to receiving your revised manuscript.

Kind regards,

Andrea Martinuzzi
Academic Editor
PLOS ONE

Journal Requirements:

Please review your reference list to ensure that it is complete and correct. If you have cited papers that have been retracted, please include the rationale for doing so in the manuscript text, or remove these references and replace them with relevant current references. Any changes to the reference list should be mentioned in the rebuttal letter that accompanies your revised manuscript. If you need to cite a retracted article, indicate the article’s retracted status in the References list and also include a citation and full reference for the retraction notice.


[Note: HTML markup is below. Please do not edit.]

Reviewers' comments:

Reviewer's Responses to Questions

**Comments to the Author**

1. If the authors have adequately addressed your comments raised in a previous round of review and you feel that this manuscript is now acceptable for publication, you may indicate that here to bypass the “Comments to the Author” section, enter your conflict of interest statement in the “Confidential to Editor” section, and submit your "Accept" recommendation.

Reviewer #1: (No Response)

Reviewer #2: (No Response)

Reviewer #3: All comments have been addressed

2. Is the manuscript technically sound, and do the data support the conclusions?

The manuscript must describe a technically sound piece of scientific research with data that supports the conclusions. Experiments must have been conducted rigorously, with appropriate controls, replication, and sample sizes. The conclusions must be drawn appropriately based on the data presented.

Reviewer #1: Partly

Reviewer #2: Yes

Reviewer #3: Yes

3. Has the statistical analysis been performed appropriately and rigorously?

Reviewer #1: Yes

Reviewer #2: Yes

Reviewer #3: Yes

4. Have the authors made all data underlying the findings in their manuscript fully available?

The [PLOS Data policy](http://www.plosone.org/static/policies.action#sharing) requires authors to make all data underlying the findings described in their manuscript fully available without restriction, with rare exception (please refer to the Data Availability Statement in the manuscript PDF file). The data should be provided as part of the manuscript or its supporting information, or deposited to a public repository. For example, in addition to summary statistics, the data points behind means, medians and variance measures should be available. If there are restrictions on publicly sharing data—e.g. participant privacy or use of data from a third party—those must be specified.

Reviewer #1: No

*Reply: The study measured the outcomes of a 12-week ecologically valid observational study of first treatment with methylphenidate in a representative clinical sample of drug naïve children with ADHD. The study was a part of the routine care in the clinic. The data were both documented in the medical record at the Child and Adolescent Mental Health Centre and in an anonymized data file with a separate key file. All data were sensitive patient information. The pseudonymous individual participant data that underlie the results reported in this article, (text, tables, figures, and appendices) can be made available to investigators for individual participant data meta-analyses that have been approved by independent review committees.*

Reviewer #2: Yes

Reviewer #3: Yes

5. Is the manuscript presented in an intelligible fashion and written in standard English?

PLOS ONE does not copyedit accepted manuscripts, so the language in submitted articles must be clear, correct, and unambiguous. Any typographical or grammatical errors should be corrected at revision, so please note any specific errors here.

Reviewer #1: Yes

Reviewer #2: Yes

Reviewer #3: Yes

6. Review Comments to the Author

Please use the space provided to explain your answers to the questions above. You may also include additional comments for the author, including concerns about dual publication, research ethics, or publication ethics. (Please upload your review as an attachment if it exceeds 20,000 characters)

Reviewer #1: (No Response)

Reviewer #2: I would suggest only a couple of points to improve the quality of the paper:
" Several meta-analyses of the short-term efficacy of immediate release MPH (IR-MPH) in randomized placebo-controlled trials
have reported large effect sizes (range from 0.54 to 1.78) on ADHD core symptoms, when effects are measured as differences
in endpoint or change in scores of parent-rated and teacher-rated ADHD symptoms and behaviours[14,15].: Network meta-analyses (eg, <https://pubmed.ncbi.nlm.nih.gov/30097390/> and <https://pubmed.ncbi.nlm.nih.gov/28700715/> ) should also be mentioned as their estimates are more precise than those form pairwise meta-analyses

*Reply: Thank you very much, the references are now added to the introduction.*

Also, two network metanalyses have reported favorable efficacy of MPH compared with placebo in randomized placebo-controlled trials[18][19].

in the discussion, the authors may refer to effects of MPH on important outcomes not generally include din RCTs, such as physical injuries (<https://pubmed.ncbi.nlm.nih.gov/31302218/>), suicides, etc...a summary is reported in <https://pubmed.ncbi.nlm.nih.gov/32905677/> .

*Reply: Again, thank you very much. The theme and references are now added to the strength and limitation section.*

The study was a short-term study whis is a limitation. The extent to which the individually monitored long-term MPH treatment can improve the prognosis is sparsely elucidated. The efficacy of pharmacological treatment of ADHD in RCTs are often using fixed doses and short follow up time periods (<six month), whereas the long-term treatment effects are limited studied[15,81]. But new long-term cohort studies have found pharmacological treatment associated to lower risk of accidents[91,92], criminality[93], suicides[94], and better academical performance[22,23,95].

Reviewer #3: Dear authors,
Congratulations on the great deal of work you have done. Your paper is original and interesting. Furthermore, the fact that there is no control group does not represent a limitation since an observational field study. Observational studies designed well and conducted according to the protocol can give the same information as experimental clinical trials. If anything, a short follow-up is unusual (12 weeks) for this type of study. Having available the administrative and clinical data of the observed cohort would be interesting to observe it for a longer period. The method used to define the predictors of the methods of treatment with methylphenidate is elegant, appropriate and very sophisticated. It is not clear the reason for choosing numerous primary objectives also since it is a multifactorial syndrome. It would be better to identify one or two main outcomes of pharmacological treatment and analyzing data in relation to these primary outcomes. In particular it is important to know the relationship between cost and benefit of treatment with methylphenidate even if there are numerous studies on this matter. Furthermore, predicters analysis is easily applicable to clinical practice? Does it allow you to change the natural history of the syndrome? In the future it would be useful to conduct a deepening also in this field. The figures and tables are clear and help in reading the paper, especially the flowchart allows you to immediately understand the design of the study.
Overall you have done a good work of a high level of statistical and a study of the factors involved in the ADHD useful for understanding the multifatorial mechanisms that are at the origin of the syndrome.
This paper does not violate the ethics code of scientific publications.

*Reply:*

*Thank you very much for the advises to review this article and for the compliments to the study.*

*It is correct that a follow-up time of 12-weeks is short-term follow-up especially in an observational study. As mentioned earlier, the current paper present data regarding the 12 weeks follow-up, while the next report from this study will focus on long-term outcome, including register-based prescription data to describe the continuation/discontinuation of MPH use over a 3-year follow-up period.*

*The purpose of this study was to detect the MPH treatment outcome (including predictors) in a broad sense with regard to the multifactorial origin, the diagnostic criteria of ADHD, and the guidelines for MPH treatment of ADHD. The aim was not to look at cost and benefit of MPH treatment, but ofcause it is an interesting research question.*

7. PLOS authors have the option to publish the peer review history of their article ([what does this mean?](https://journals.plos.org/plosone/s/editorial-and-peer-review-process#loc-peer-review-history)). If published, this will include your full peer review and any attached files.


If you choose “no”, your identity will remain anonymous but your review may still be made public.


**Do you want your identity to be public for this peer review?** For information about this choice, including consent withdrawal, please see our [Privacy Policy](https://www.plos.org/privacy-policy).

Reviewer #1: No

Reviewer #2: No

Reviewer #3: No

[NOTE: If reviewer comments were submitted as an attachment file, they will be attached to this email and accessible via the submission site. Please log into your account, locate the manuscript record, and check for the action link "View Attachments". If this link does not appear, there are no attachment files.]

While revising your submission, please upload your figure files to the Preflight Analysis and Conversion Engine (PACE) digital diagnostic tool, <https://pacev2.apexcovantage.com/>. PACE helps ensure that figures meet PLOS requirements. To use PACE, you must first register as a user. Registration is free. Then, login and navigate to the UPLOAD tab, where you will find detailed instructions on how to use the tool. If you encounter any issues or have any questions when using PACE, please email PLOS at [figures@plos.org](mailto:figures@plos.org). Please note that Supporting Information files do not need this step.
